# Understanding professional disparities in academic anesthesiology: a single-center gender-based survey study

**DOI:** 10.1186/s12871-025-03522-z

**Published:** 2025-12-07

**Authors:** Anne L. Donovan, Joyce Chang, Jina Sinskey, Rachel Schwartz

**Affiliations:** 1 Department of Anesthesia & Perioperative Care, University of California, San Francisco, 505 Parnassus Avenue, San Francisco, USA; 2 Division of General Internal Medicine, University of California, San Francisco, San Francisco, USA

**Keywords:** Women in academic anesthesiology, Gender equity, Academic success, Advancement and promotion, Career longevity, Intent to leave, Work-life integration, Work culture, Mentorship, Sponsorship

## Abstract

**Background:**

Women in academic anesthesiology face numerous structural and cultural barriers that impede academic advancement and threaten career longevity. Understanding the impact of these challenges can allow steps to be taken to improve gender equity and retention in academic anesthesiology.

**Methods:**

This study describes a voluntary, anonymous survey distributed to faculty members in the Department of Anesthesia & Perioperative Care at an urban tertiary care academic Health System with a variety of anesthetizing locations across six hospitals. All clinical faculty members in the Department, regardless of gender identity, were invited to participate (*n* = 209). The 57-question survey, which was administered over a 3-week time period in 2024, examined the relationship between demographic variables and professional factors relating to career progression. Quantitative data were summarized with descriptive statistics to compare responses between both gender and career stage peer groups. Using inductive thematic analysis, the authors analyzed open-ended survey data and developed themes that will be used to develop future improvement initiatives.

**Results:**

The survey response rate was 35% (73/209). Respondents were well-balanced in terms of gender (52.1% women, 45.2% men, 2.7% other) and other demographic groups. Women respondents more often remained at an assistant professor level after 6–10 years of practice (36.4% vs. 0%), reported fewer first or senior author publications if in practice 5 + years (70% vs. 33.3%), and described non-promotable work responsibilities in early- and mid-career time points (85% vs. 67%). Women less often reported receiving an accelerated promotion (33.3% vs. 44.8%); holding departmental (50% vs. 58.6%), institutional (17.9% vs. 35.7%), or national (34.6% vs. 44.8%) leadership positions; and having participated on an editorial board (26.5% vs. 37.9%). More women, particularly in early- and mid-career stages, reported active intent to leave the institution (21.1% vs. 12.1%). Three clear themes emerged from qualitative survey data: 1) Career Advancement, 2) Work Culture, and 3) Work-life Integration.

**Conclusions:**

Differences are reported in traditional metrics of academic career success between men and women in an academic anesthesiology department. The themes identified provide concrete targets for improvement in improving equity between women and men and retention in academic anesthesiology.

**Supplementary Information:**

The online version contains supplementary material available at 10.1186/s12871-025-03522-z.

## Background

Widespread physician shortages threaten patient care delivery and contribute to physician burnout. Anesthesiologists report the highest rate of intention to leave academic medicine at 46.8% [1]. While women account for over 50% of matriculating medical school students [2], only 26.5% of all US anesthesiologists are women [3]. Factors that threaten the retention of women in academic medicine include lower likelihood to be promoted to higher academic ranks or to leadership roles [4] and gaps in traditional measures of academic success such as the number of publications [5], compensation [6], and citation of research [7]. Gender-based differences in workplace culture also contribute to burnout and attrition [8]. Women reported both a lower rate of professional fulfillment and a higher rate of burnout than men in a recent cross-sectional survey of academic physicians from 15 academic medical institutions [1]. Similar factors that threaten the retention of women anesthesiologists are present at both departmental and professional organizational levels [9–17]. Issues that disproportionately affect women anesthesiologists include harassment and mistreatment in the perioperative environment [17–20], structural barriers preventing promotion to leadership positions [11–15, 21–23], and the coexisting demands of clinical and academic work with parenthood and other personal obligations [15–17, 20–22, 24–27].

The current shortage in physician anesthesiologists is likely to increase in the coming years [28]. Strain on the anesthesia workforce, particularly after the COVID-19 pandemic, threatens to adversely affect the quality and cost of patient care, worsen provider burnout, and diminish the feasibility of accomplishing the academic anesthesiology mission [1, 29]. Turnover of faculty in academic departments also increases expense in recruiting, hiring, and training new faculty members [30]. The combination of workforce scarcity and retention challenges creates an existential threat to academic anesthesiology; however, developing new strategies to improve retention of women academic anesthesiologists is an actionable goal.

While identifying and supporting high-risk individuals has been proposed as a strategy to improve the retention of women anesthesiologists [17, 31, 32], there are few concrete examples in the literature of such initiatives. This initiative, LAADIES (Leveling Academic Anesthesiology Disparities and Inequities to Encourage Success) sought to first characterize the current work environment for women faculty members, then to develop and implement interventions to improve retention and well-being of women academic anesthesiologists. Here, descriptive and qualitative data are presented on the initial phases: identification of problem areas and conception of retention interventions.

## Methods

A 57-question survey instrument was developed to gather information on demographics, career path, leadership roles and productivity, academic productivity, promotion, mentorship, intent to stay, well-being, parenthood, and general perceptions. Questions were designed to investigate common career barriers for women in academic anesthesiology both nationally and locally identified through literature search, Health System Net Promoter Score survey data, departmental wellness data, and lived experiences of the authors. Face validity of survey items was ensured by two of the authors (A.D., an established-career critical care physician and J.C., a mid-career critical care physician), and content validity was evaluated by the remaining two authors (R.S., an early-career health services researcher with expertise in qualitative research methods and well-being research and J.S., a mid-career anesthesiologist with expertise in well-being research) to ensure that survey questions adequately measured the desired construct. Using the survey instrument, the relationship between anesthesiologist demographic variables and professional factors relating to career progression was examined (See Supplemental Survey Document).

The survey was distributed via e-mail to all clinical faculty members in departmental e-mail distribution lists (*n* = 209) from the Department of Anesthesia & Perioperative Care at an urban academic tertiary care health system during a three-week period in May–June 2024. The health system includes five distinct hospital locations and a Level 1 county trauma hospital, which collectively provide anesthesia care to a diverse range of patients of all ages, socioeconomic status, acuity, and surgical subspecialty populations, in both inpatient and ambulatory practice settings. The survey took approximately 15–20 min to complete. Survey data were collected using Qualtrics software (Qualtrics, Provo, UT), a platform available only through the institution’s virtual private network that uses triple layer security encryption and password protection. Although the survey was designed to improve understanding of the experience of women anesthesiologists, the survey was distributed to all faculty members to allow for gender-based comparisons during the analysis. Participation in the survey was voluntary and anonymous, and subjects provided informed consent on the survey’s opening page by electing to proceed with the survey (Appendix 1 of Supplemental Document 1). At the end of the survey, study participants were offered the voluntary opportunity to provide their email address in a separate form that was de-linked from survey responses (Appendix 2 of Supplemental Document 1). Four study participants chosen at random received a gift card as an incentive for participation. Selection of gift card recipients was overseen by the study first author (A.D.).

After the initial email survey invitation, a reminder email was sent on weeks 2 and 3 of the study. Few additional survey responses were received after the second survey reminder email was sent, so the survey was closed after week 3. Additionally at this time, new survey responses revealed similar quantitative patterns and qualitative themes. No a priori sample size calculation was performed. This study was granted exempt status by the institution’s Institutional Review Board (IRB # 23–39,253). Artificial intelligence was not used in any part of conducting this study or in preparation of the manuscript.

### Statistical analysis

Quantitative data categories collected are listed in Table 1. Variables were summarized with descriptive statistics, including frequency count and percentage for categorical variables. Respondents were not required to answer all survey questions; missing responses were not counted and any data received in submitted surveys was used for analysis. Instructions provided to survey participants are available in Appendices 1 and 2 of Supplemental Document 1. Since the survey was anonymous, it was not possible to track characteristics of non-responders.

**Table 1:** Quantitative survey data

Quantitative data category	Definition (when applicable)
Participant demographics	
Percent effort in professional activities	
Academic rank	
Success at promotion	
Leadership roles	Director or Associate Director, Chair or Vice Chair, Editor or Associate Editor, appointed/elected committee assignments, or leadership in a professional organization
Editorial board membership	
Publications in the past 5 years	First, second, or senior author
Research funding	
Awards received	
Non-promotable activities	
Allyship, mentorship, sponsorship	Ally: “*a trusted person who supports you and helps you achieve your goals*” Mentor: “*an advisor who offers advice and/or guidance on career and work, often based on their own experience”* Sponsor:* “a person who uses their own influence or social capital to create or connect you with career-advancing opportunities”*
Parenting & caregiving responsibilities	
Impostor phenomenon	*“A persistent doubt of one’s own abilities and skills (i.e., that your own abilities are not equal to those around you) and fear of being discovered as a ‘professional fraud’”*

Open-ended survey responses were compiled in Microsoft Excel and analyzed using an inductive thematic analysis approach [33] and a constructivist paradigm. The coding team consisted of three authors: A.D, J.C., and R.S. Each independently reviewed all responses and developed a set of initial codes, then met together to align their codes into a single codebook by consensus (see Supplemental Document 2 for codebook), They then re-coded all qualitative data by applying agreed-upon emergent codes and comparing their coding to ensure intercoder reliability. Any discrepancies in coding were resolved by consensus. Emerging themes were triangulated with quantitative responses from the survey.

The research team employed reflexivity to mitigate the potential impact of researchers’ personal and professional biases. Having a non-clinical health services researcher (R.S.) in the study team served to provide an “outsider” perspective that further enriched the data analyses. A Standards for Reporting Qualitative Research (SRQR) checklist was used to describe the qualitative data [34].

## Results

Survey response rate was 35% (73/209). Demographic information is summarized in Table 2. 38 (52.1%) respondents were women, 33 (45.2%) were men, one (1.4%) was non-binary/gender fluid, and one (1.4%) did not disclose their gender identity. Since the number of respondents with gender identity other than man or woman was low, only responses from those identifying as woman or man were included for analysis. When compared with the gender make-up of the department (42.0% women, 50.2% men, 7.7% unknown gender), based on human resources data available at UCSF Health in July 2024, women are slightly overrepresented in the survey. Responses were compared between both gender and career stage peer groups. Career stages were defined by number of years on faculty (0–5 years, 6–10 years, 11–15 years, 16–20 years, 20 + years). Where further detailed, early-career was defined as 0–5 years on faculty, mid-career 6–15 years, and established-career 16 or more years.

**Table 2:** Demographics of Survey Participants

Category	Characteristic	Count	%
Gender Identity	Male	33	45.2%
	Female	38	52.1%
	Non-binary/gender fluid	1	1.4%
	Prefer not to answer	1	1.4%
Age group	30–39	20	27.4%
	40–49	29	39.7%
	50–59	14	19.2%
	60–69	7	9.6%
	70 +	3	4.1%
Years in Practice	0–5	29	40.3%
	6–10	17	23.6%
	11–15	11	15.3%
	16–20	8	11.1%
	> 20	7	9.7%
Academic Title	Clinical Instructor	4	5.6%
	Assistant Professor	20	27.8%
	Associate Professor	24	33.3%
	Professor	22	30.6%
	Other	2	2.8%
Race/ethnicity	Asian or Pacific Islander	21	28.8%
	Black or African American	3	4.1%
	Hispanic/Latino(a)	1	1.4%
	White/Caucasian	35	47.9%
	Multiple ethnicity/Other not listed	10	13.7%
	Prefer not to say	3	4.1%
Member of group underrepresented in medicine	Underrepresented racial minority	10	13.7%
	Non-native English speaker	10	13.7%
	Prefer not to answer	8	11.0%
	Underrepresented religious minority	6	8.2%
	Member of the LGBTQIA + Community	4	5.5%
	Other	3	4.1%
Marital/partnership status	Single/never married	6	8.2%
	Married or domestic partnership	61	83.6%
	Divorced	3	4.1%
	Prefer not to say	3	4.1%
Parent	Yes	46	76.7%
	No	14	23.3%
Subspecialty practice	Acute pain	7	9.7%
	Airway	6	8.3%
	Ambulatory surgery	2	2.8%
	Cardiac	3	4.2%
	Chronic pain	6	8.3%
	Critical care	13	18.1%
	Liver transplant	4	5.6%
	Neurosurgery	6	8.3%
	Obstetric anesthesia	5	6.9%
	Other	3	4.2%
	Pedi cardiac	4	5.6%
	Pediatrics	7	9.7%
	Prefer not to say	7	9.7%
	Preop evaluation clinic	1	1.4%
	Regional	10	13.9%
	Spine	8	11.1%
	Thoracic	4	5.6%
	Vascular	5	6.9%
	Average teams per respondent	1.38	
Average % Effort	Clinical work		58.9%
	Research/creative activities		27.9%
	Education		13.8%
	Clinical operations		14.3%

### Quantitative survey results

Selected results are summarized below and listed in Table 3. Detailed results can be found in Supplemental Tables and Figures in Supplemental Document 1.

**Table 3:** Summary of Qualitative Survey Data

Category	Data Summary	Women [n (%)]	Men [n (%)]
Academic Rank	Assistant professor rank after 6–10 years on faculty	4/11 (36.4%)	0/6 (0%)
	Full professor rank at 11–15 years on faculty	4/5 (80%)	1/5 (20%)
	Full professor rank at 16–20 years on faculty	6/6 (100%)	9/9 (100%)
Promotion	Accelerated promotion (any career timepoint)^a^	11/33 (33.3%)	13/29 (44.8%)
	Denied an on-time promotion^a^	1/33 (3.0%)	4/29 (13.7%)
Leadership Roles	Departmental leadership positions^a^	17/34 (50%)	17/29 (58.6%)
	Departmental leadership position < 10 years on faculty	11/25 (44%)	10/16 (62.5%)
	Institutional leadership positions^a^	5/28 (17.9%)	10/28 (35.7%)
	National leadership positions^a^	9/26 (34.6%)	13/29 (44.8%)
	National leadership position 11 + years on faculty	2/8 (25%)	8/13 (61.5%)
Editorial Board Participation	Ever served on editorial board^a^	9/34 (26.5%)	11/29 (37.9%)
	Ever served on editorial board (by years on faculty)	0-5y: 1/14 (7.1%)	0-5y: 1/11 (9.1%)
		6-10y: 6/11 (54.5%)	6-10y: 3/5 (60%)
		11-15y: 1/3 (33.3%)	11-15y: 2/5 (40%)
		16-20y: 1/4 (25%)	16-20y: 2/3 (66.7%)
		> 20y: 0/2 (0%)	> 20y: 3/5 (60%)
Publications	0–2 publications > 5 yr on faculty	14/20 (70%)	6/18 (33%)
	3 + publications > 5 yr on faculty	6/20 (30%)	12/18 (66.7%)
Non-Promotable Activities	Non-promotable responsibilities </= 15 yr on faculty	23/27 (85%)	14/21 (67%)
	Non-promotable responsibilities > 20 yr on faculty	3/5 (60%)	8/8 (100%)
Allyship, Mentorship, Sponsorship	At least one ally^a^	27/34 (84.3%)	21/27 (77.8%)
	Five or more allies^a^	12/27 (44.4%)	11/21 (52.4%)
	At least one mentor^a^	23/32 (71.9%)	17/26 (65.4%)
	One or two mentors^a^	20/23 (87.0%)	12/17 (70.6%)
	At least one sponsor^a^	18/31 (58%)	11/26 (42.3%)
	One sponsor^a^	12/18 (66.7%)	7/11 (63.6%)
Intent to Leave	Active intent to leave the institution^a^	8/38 (21.1%)	4/33 (12.1%)
	Considering another academic dept^a^	3/8 (37.5%)	1/4 (25%)
	Considering private practice^a^	5/8 (62.5%)	1/4 (25%)
	Considering leaving medicine entirely^a^	0/8 (0%)	2/4 (50%)
	Considering industry^a^	1/8 (12.5%)	0/4 (0%)
	Considering “other”^a^	1/8 (12.5%)	0/4 (0%)
Parenting	Primary caregiver for children^a^	8/26 (30.7%)	0/18 (0%)
	Primary parent^a^	11/14 (78.6%)	2/10 (20%)
	Primary parent on faculty 0–5 years	4/7 (57.1%)	1/2 (50%)
	Primary parent on faculty 6 + years	7/7 (100%)	1/6 (16.7%)
	4 + hours/week coordinating childcare^a^	9/24 (37.5%)	3/17 (17.6%)
Impostor phenomenon	Experience to some extent, a great extent, or constantly^a^	25/33 (75.8%)	16/28 (57.1%)
	Experience to some extent, a great extent, or constantly 0–5 years on faculty	10/14 (71.4%)	7/10 (70%)
	Experience to some extent, a great extent, or constantly 20 + years on faculty	2/2 (100%)	2/5 (40%)

^a^denotes data refers to all career stages combined

#### Career advancement

##### Academic rank

**Fig. 1:**
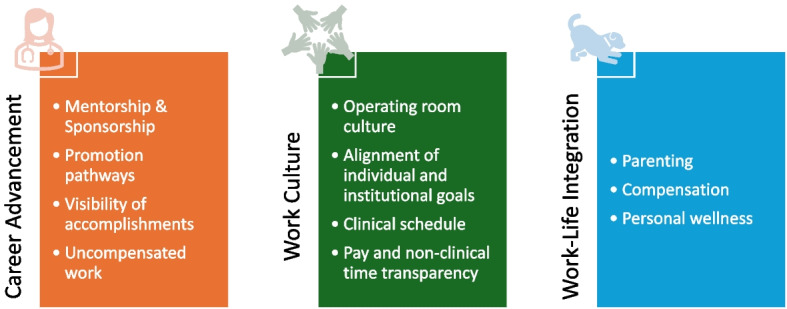
Themes and Sub-themes Arising from Qualitative Data Analysis. Three clear themes (vertical text) were identified using qualitative data analysis. Sub-themes are listed in bullet points in the solid boxes

Survey respondents were representative of the department in terms of academic rank based on available human resources data as of July 2024 (38.7% Assistant Professor, 27.6% Associate Professor, 33.7% Professor). Compared with men, more women remained at an assistant professor rank after 6–10 years on faculty. At 11–15 years on faculty, more women had advanced to full professor (Table 3, Supplemental Table and Fig. 1).

##### Promotion

Men received an accelerated promotion more frequently than women, but were more often denied an on-time promotion at any career timepoint (Table 3). For men, the most common time point for failed promotion was 11–15 years (3/4, 75%).

##### Leadership roles

**Fig. 2:**
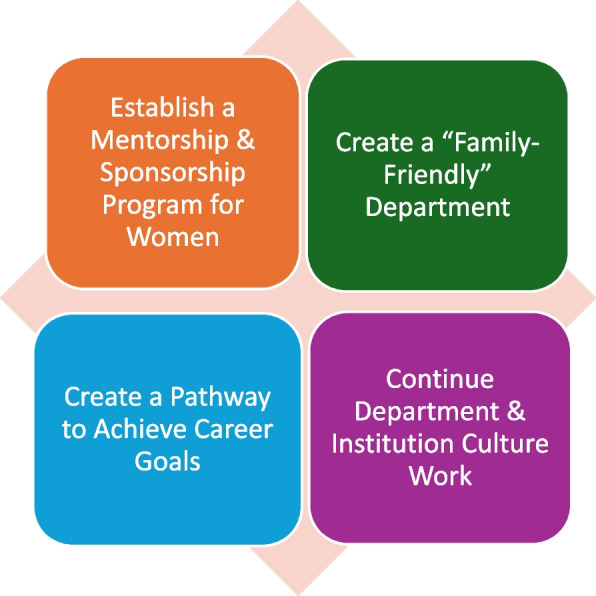
Priority Areas for Future Intervention. The four broad areas we intend to focus on for improvement based on survey data analysis are: establish a mentorship and sponsorship program for women, create a “family-friendly” department, create a pathway to achieve career goals, and continue ongoing departmental and institutional work aimed at improving work culture

Proportionally more men than women held leadership positions at the departmental, institutional, and national levels overall (Table 3). Early-career men held a departmental leadership position more often than women, and mid- to established-career men more often held a national leadership position. At multiple time points, men more commonly held an institutional (extra-departmental) leadership position than women (Supplemental Tables and Figs.2–4).

##### Editorial board participation

Women less frequently reported ever having been asked to serve as a member of an editorial board overall and at all time points throughout their careers (Table 3, Supplemental Table and Fig. 5).

##### Publications

Women within their first 5 years on faculty more often reported 3 or more first, second, or senior author publications than their male peers; however, among those on faculty for five or more years, men more frequently reported publishing 3 or more while women more often reported 0–2 publications in these roles (Supplemental Fig. 6 and Tables 6.1, 6.2).

##### Research funding and awards

At specific career time points, women less often reported ever receiving extramural research funding (i.e., 0–5 years, 6–10 years, and 16–20 years) and intramural research funding (i.e., 0–5 years, 11–15 years, and 16–20 years) (Supplemental Table and Fig. 7.1, 7.2) (Supplemental Tables and Figs. 8 and 9).

##### Non-promotable activities

Early- and mid-career women (i.e., those on faculty for 15 years or less) more often reported having non-promotable work responsibilities (e.g., social event planning), whereas established-career men (i.e., on faculty for 16 years or more) more frequently reported participation in non-promotable activities than established-career women (Table 3, Supplemental Table and Fig. 10).

##### Allyship, mentorship, sponsorship

Overall, women reported having at least one ally, mentor, and/or sponsor in the department more frequently than men. Definitions of these terms is provided in Table 1 and the Survey Supplement. Of those with allies, the most commonly reported number of allies was five or more; of those with mentors, the most commonly reported number of mentors was one or two; and of those with sponsors, the most commonly reported number of sponsors was one (Table 3, Supplemental Table 11).

#### Intent to leave

More women than men indicated they were actively considering leaving the institution. Of the 8 women considering leaving, 2 were early-career, 4 were mid-career, and 2 were established-career. Of the 4 men considering leaving, 3 were early-career and one was established-carer. Respondents were considering another academic department, private practice, leaving medicine entirely, industry, or other. Reasons cited for leaving or staying are listed in Supplemental Table 12.

#### Parenting

Women parents more often identified themselves as the primary caregiver of their children and spending at least four hours per week coordinating care for their children. All mid-career women parents (7/7) reported being the primary parent, compared with only 1/6 (16.7%) mid-career men (Table 3, Supplemental Fig. 12). Sources of stress for parents are discussed in Supplemental Table 13, and include childcare, coordination of care, presence/contribution, clinical schedule, and lactation. When asked whether parenting stressors are different for women and men, 28/44 respondents (63.7%) answered probably or definitely yes. Of those respondents answering “definitely yes,” 13/19 (68.4%) were women; of those respondents answering “definitely no,” none were women (Supplemental Fig. 13).

#### Impostor phenomenon

Overall, women reported experiencing impostor phenomenon to some extent, a great extent, or constantly more frequently than men. Both men and women on faculty for 0–5 years were most likely to report at least some extent of impostor phenomenon. Both men and women on faculty for 20 or more years reported experiencing impostor phenomenon to at least some extent, though it was more commonly reported in established-career women faculty [women: 2/2 (100%) vs. men 2/5 (40%)]. Of note, women who experienced impostor phenomenon described it as a barrier or a setback to their day-to-day clinical and academic work in open-ended questions, whereas men mostly described impostor phenomenon as a motivating factor or a mechanism to prevent overconfidence (Supplemental Table and Supplemental Fig. 14).

### Qualitative survey results

398 comments were received to 18 open-ended questions. Three clear themes emerged from qualitative survey data: 1) Career Advancement, 2) Work Culture, and 3) Work-life Integration (Fig. 1). Selected quotes illustrating these themes are described in Supplemental Table 15.

#### Career advancement

##### Challenges

This theme highlights multifactorial challenges women face to attain leadership positions and advance their careers. Lack of mentorship and sponsorship, lack of a clear path to advancement and retention, and leadership positions with significant off-duty hours expectations are a few of the issues further detailed in Supplemental Table 15.

##### Proposed solutions

Identified sub-themes highlight the need to develop individualized initiatives targeted to advancing women’s academic careers (Fig. 1). Creation of a structured *mentorship and sponsorship* program for every woman faculty member; clarifying the *promotion pathways for women* while creating opportunities for early-career women to help themselves establish a path to academic success; enhancing the *visibility of accomplishments* by advancing women to highly visible leadership positions and also by elevating women faculty’s success to an equal extent as men’s; and reducing the burden of *uncompensated work* on women faculty members are suggested solutions (Supplemental Fig. 15).

#### Work culture

##### Challenges

Both women and men faculty members highlighted cultural issues which negatively impact their sense of well-being, belonging, and accomplishment in their work environment, many of which have a disproportionate impact on women. Four sub-themes were identified: *operating room (OR) culture*, *alignment between institutional and individual values, clinical schedule, and pay and non-clinical time transparency*. Microaggressions and disrespect toward women and individuals from underrepresented groups were frequently discussed in open-ended questions, as were lack of community and poor teamwork and communication. Perceptions of institutional priorities differing from those of providers and misalignment between stated and perceived institutional priorities also arose as prevalent challenges. The need for scheduling procedures that align with faculty needs was also discussed, as was fairness and transparency in allocation of non-clinical time and leadership roles.

##### Proposed solutions

Work culture challenges may be addressed by improving the culture of teamwork, efficiency, and mutual respect in the OR environment; working with the institution and department to align toward the long-term goals that resonate with faculty members; optimizing shift timing, schedule predictability, and end of day relief to balance the demands of work and home; and improving the transparency of non-clinical time allocation and pay differentials between faculty members (Supplemental Fig. 16).

#### Work-life integration

##### Challenges

Work-life integration is a challenge which permeated the other identified themes. Respondents identified the need to create an appropriate balance between the demands at work and one’s responsibilities or interests outside of work. Many of the identified challenges arose from *parenthood* responsibilities, but *compensation* and *personal wellness* were also raised as specific concerns*.* Within the parenting sub-theme, respondents experience a significant amount of tension between parenthood and academic success (Supplemental Table 13).

##### Proposed solutions

Potential solutions for parents include accommodation of flexible work arrangements, providing lactation accommodations, improving the process of return to work from parental leave, assisting with availability and affordability of childcare, and making work conducive to presence/quality time with family (Supplemental Fig. 17). To address compensation, matching income with the high cost of living in the institution’s area is needed. Personal wellness can be improved by efforts to improve faculty members’ time and energy to pursue their interests outside of the workplace.

## Discussion

This study suggests that women in an academic anesthesiology practice may lag behind men in their department in traditional metrics of academic career success including leadership positions, grant funding, publications, and editorial board service. More women remained at lower academic ranks earlier in their careers, and more men received accelerated promotions. Women expressed intent to leave at a higher frequency then men and more frequently report primary caregiving responsibilities for their children. Barriers potentially impeding women’s career advancement and threatening career longevity in academic medicine and outline potential solutions are explored here.

The study’s qualitative findings align with prior studies describing contributors to this problem, including lack of mentorship and sponsorship, parenthood demands, inflexible scheduling processes, excess burden of non-promotable work, structural bias, paucity of women role models, and differences in career values between women and men [19, 20, 35]. The impact of microaggressions, mistreatment, marginalization, and workplace practices that create a hostile environment for women [17, 19, 20, 25, 36] also plays a role. These factors likely contribute significantly to anesthesiologists reporting the highest intent to leave among all subspecialties evaluated in a multi-institution survey [1]. It is notable that both men and women in the study identified professional concerns, and male respondents advocated for interventions and programs specifically for women. In line with existing literature, the authors identified themes of *Career Advancement*, *Work Culture*, and *Work-life Integration* from qualitative data.

This study demonstrated a pattern, which the authors will call the “mid-career switch,” where more early-career women remained at a lower academic rank and had fewer academic achievements such as grant funding, but at the mid-career stage (i.e., 11 or more years) more women than men had been promoted to full professor and also had more academic achievements including departmental and institutional leadership positions, extramural research funding, and national awards. Further, more men failed promotion at the 11–15 year mark than women. This pattern could be explained by attrition of early- and mid-career women who did not see a path to advancement or success in academic medicine, a phenomenon which has been described as a “leaky pipeline” by other authors [37]. The authors did not have access to human resources data to confirm higher attrition rates in women, but data related to intent to leave provides additional support for this theory. More women than men (21.1% vs. 12.1%) indicated their active intent to leave, and most women considering leaving the institution were within the first 10 years of their careers. This would suggest that women who are not yet “established” in their careers are at risk of leaving academic medicine, perhaps at higher rates than men. Parenthood data may provide further insight into these findings, as 100% of women parents on faculty for 6 or more years described themselves as the primary parent, compared with 0–33.3% of men. Women in general, and women parents in particular, may be drawn to leave academic medicine for better work-life integration, higher compensation, improved schedule flexibility and predictability, reduced cognitive load, and escape from mistreatment, among other reasons.

*Mentorship and sponsorship* is a sub-theme of the *Career Advancement* theme in this study. Prior studies have shown that mentorship and sponsorship improve women’s likelihood of success in promotion and leadership [31, 32]. Mentorship and sponsorship for early-career women may address some of the factors that lead to attrition by helping them to achieve their career goals and find a sustainable path in academic medicine. To make strides toward this goal in the department, authors are developing a structured faculty mentorship and sponsorship program with components targeted to women. By creating career development opportunities for early- and mid-career women, the goal is to improve academic success and retention (Fig. 2).

*Uncompensated work* and *Parenting* are two additional interrelated sub-themes in this study. It is well-described that women disproportionately hold non-promotable roles both at work (e.g., social event planning, colleague support) and at home (e.g., parenting, household chores) [38]. In this study, early-career women faculty assumed responsibility for non-promotable professional activities more frequently than men, and women parents report more primary parenting responsibilities and time coordinating care for their children. These differences were particularly notable during mid-career, consistent with prior reports emphasizing the impact of delayed childbearing resulting in increased parenthood demands on mid-career women [20]. Parents identified the need for improved processes for transition back to work after parental leave, more flexible scheduling options, schedule predictability, and accommodations for lactation, also consistent with existing literature [24]. There is a need to design leadership and academic opportunities that respect time with family and parental responsibilities [39]. Other authors [39] recently called for a change in both our expectations in academic medicine and in the way we work to ensure that people are given equal opportunities, regardless of gender. Acknowledging that women are more likely primary caregivers for their families and that this work requires substantial time and emotional energy, suggested changes include de-emphasizing professional opportunities requiring significant travel, allowing more remote meetings and presentations to encourage participation of those unable to travel, and promotion based on a holistic evaluation of one’s accomplishments in the context of one’s full personal circumstances. In response, the authors have set the goal of creating a “Family-friendly” department (Fig. 2). While implementation of this goal is under study, recommendations made by other authors [39] serve as a starting point.

The theme *Work Culture* highlights issues linked to burnout among healthcare workers [40]. Problems such as microaggressions and mistreatment of individuals from underrepresented groups, lack of teamwork and collaboration, fairness of resource allocation, lack of flexibility and predictability in clinical scheduling, and excessive workload are challenging to overcome but nonetheless represent a threat to the anesthesiology workforce. Therefore, urgent attention to improving work culture is necessary (Fig. 2).

This study has several limitations. First, the intention of the quantitative data presented was purely to provide descriptive information to complement the qualitative portion of the study. Since it was not designed to yield statistically significant quantitative comparisons, no statistical measures are reported. Quantitative results reported should thus be interpreted as hypothesis-generating.

The single health system sampled may limit generalizability of data, as the authors’ institution has specific promotion requirements (i.e., assessment of achievement and promise in teaching/mentoring, research/creative work, professional competence, and University/public service) that may differ from other institutions. Further, compared with the US anesthesia workforce [3], the faculty is composed of significantly more women (42% vs. 26.5%). The department has an infrastructure for well-being and diversity, equity, and inclusion that may have influenced faculty members’ experiences and perspectives. However, generalizability is improved by the multi-site health system consisting of six diverse clinical sites that vary in terms of patient population, socioeconomic status, clinical acuity, medical complexity, case mix, and subspecialty population in which it was conducted, since the diversity of practice of clinicians sampled in this survey makes the sample more representative of anesthesiologists working in varying practice settings. In addition, survey respondents were demographically diverse and, with the exception of a higher proportional sample of women in the study, were generally representative of the department makeup to the extent that data could be compared.

The privacy restrictions to Human Resources data describing demographics in the department limited authors’ ability to compare the study population to the broader department except for gender and rank, as well as to understand the impact of attrition rates on the study’s findings. The 35% survey response rate is within range of other surveys published in the anesthesiology literature [41]; however a higher response rate was likely not achieved due to an already high burden of clinical and administrative tasks facing faculty members. We believe the sample size is sufficient to generally characterize the environment in academic anesthesiology, though local differences may exist.

This study adds to the literature describing the barriers for women in academic anesthesiology and begins to outline concrete areas for improvement. Several recent studies report increasing gender equity in anesthesiology [15, 16, 21, 22, 26, 27]; the authors hope to contribute to this momentum with ongoing awareness and targeted interventions. Based on this study and with additional data currently being collected, the department plans to take numerous steps to encourage academic success for women faculty (Fig. 2). Such steps should be encouraged more broadly to promote the academic success and retention of women anesthesiologists to respond proactively to projected shortages in the anesthesiology workforce.

## Conclusions

Differences may exist in traditional metrics of academic career success between men and women at a large and well-respected Anesthesiology department with robust infrastructure for wellness and diversity, equity, and inclusion. There are many contributors to this finding, centering around three themes identified: *Career Advancement*, *Work-life Integration*, and *Work Culture*. Within the identified themes, concrete areas emerged as the focus of improvement strategies that aim to advance gender equity and retention of women in academic anesthesiology.

## Supplementary Information

Supplementary Material 1: Survey Supplement: Survey instrument.

Supplementary Material 2: Supplemental Document 1: Detailed Survey Results. Quantitative Survey Results. Supplemental Figure and Table 1: Academic rank by years on faculty. Supplemental Figure and Table 2: Department leadership positions by years on faculty. Supplemental Figure and Table 3: Institutional leadership positions by years on faculty. Supplemental Figure and Table 4: National leadership positions by years on faculty. Supplemental Figure and Table 5: Ever served on an editorial board by years on faculty. Supplemental Figure and Table 6: Number of publications in the past 5 years by years on faculty. Supplemental Table 6.1: 0-2 vs. 3+ publications by years on faculty. Supplemental Figure and Table 7.1 and 7.2: Research funding by years on faculty. Supplemental Figure and Table 8: Number of Departmental Awards by Years on Faculty. Supplemental Figure and Table 9: Number of National Awards by Years on Faculty. Supplemental Figure and Table 10: Non-promotable activity by years on faculty. Supplemental Table 11: Allies, Mentors, and Sponsors. Supplemental Table 12: Reasons for Leaving or Staying. Supplemental Figure 12: Primary parent. Supplemental Table 13: Sources of stress for parents. Supplemental Figure 13: Parenting stress differences between men and women. Supplemental Figure and Table 14: Impostor phenomenon. Qualitative Survey Results. Supplemental Table 15: Representative quotes related to survey themes and sub-themes. Supplemental Figure 15: Potential Interventions to Address Career Advancement Sub-themes. Supplemental Figure 16: Potential Interventions to Address Work Culture Sub-themes. Supplemental Figure 17: Potential Solutions for Providing Balance between Home and Work for Parents

Supplementary Material 3: Supplemental Document 2: Codebook

## Data Availability

Data is provided within the manuscript or supplementary information files.

## Abbreviations

LAADIES: Leveling Academic Anesthesiology Disparities and Inequities to Encourage Success
IRB: Institutional Review Board
SRQR: Standards for Reporting Qualitative Research
OR: Operating room
