# Supplementary material for: Understanding professional disparities in academic anesthesiology: a single-center gender-based survey study
Source: BMC Anesthesiol. 2025 Dec 7;26:31. doi: 10.1186/s12871-025-03522-z (PMC12797490; doi:10.1186/s12871-025-03522-z)
Supplement: Supplementary file 1 — Supplementary Material 1: Survey Supplement: Survey instrument. [file 12871_2025_3522_MOESM1_ESM.pdf]

Demographics

The following questions about demographics are essential to helping us better understand how career progress and work experience are influenced by participants' intersecting identities. Please answer to the best of your ability.

1. Which most closely describes your gender?
   Male, Female, Non-binary / gender fluid, Transgender woman, Transgender man, Other (please specify), Prefer not to answer

2. Which race or ethnicity best describes you?
   American Indian or Alaskan Native, Asian/Pacific Islander, Black or African American, Hispanic / Latino(a), White/Caucasian, Multiple ethnicity/Other (please specify), Prefer not to say

3. In which age group do you belong?
   20-29, 30-39, 40-49, 50-59, 60-69, 70+

4. Are you a member of un underrepresented or vulnerable group in anesthesia? If yes, please choose all that apply. If no, please skip.
   Underrepresented racial minority (please specify – optional)
   Underrepresented religious minority (please specify – optional)
   Member of the LGBTQIA+ Community (please specify – optional)
   Non-native English speaker
   Other – please specify (optional)
   Prefer not to answer

5. What is your marital/partnership status?
   Single/never married, married or domestic partnership, widowed, divorced, separated, prefer not to say, other (please specify)

6. In what ways do your personal identities impact your experience at work?

Career path and interests

7. Where is your primary work location?
   UCSF Health (i.e., Parnassus, MB, MZ, POSC, OI, etc.), ZSFG, Other

8. Do you belong to any of the following sub-specialty clinical teams? Select all that apply or skip if not applicable.
   Neuroanesthesia, spine, vascular, thoracic, airway, regional, acute pain, chronic pain, OB, critical care, cardiac, liver transplant, pediatrics, pedi cardiac, preop evaluation clinic, ambulatory surgery, other (please specify), prefer not to say

9. How many years have you been on faculty in this department?
   0-5, 6-10, 11-15, 16-20, > 20

10. What is your Title?
    Clinical instructor, Assistant Professor, Associate Professor, Professor, other (please specify)

11. What percent effort do you currently spend on the following activities:

	0%	1-19%	20-39%	40-59%	60-79%	80-100%
Clinical work	☐	☐	☐	☐	☐	☐
Research/creative work	☐	☐	☐	☐	☐	☐
Education	☐	☐	☐	☐	☐	☐
Clinical operations	☐	☐	☐	☐	☐	☐
Other (specify)	☐	☐	☐	☐	☐	☐

Leadership Roles and Academic Productivity

Leadership roles for the purpose of this survey are defined as roles with the title (or a similar/equivalent title): Director or Associate Director, Chair or Vice Chair, Editor or Associate Editor, and/or appointed/elected committee assignments, or leadership in a professional organization.

12. How many of each type of leadership role do you/have you held?

	0	1	2	3	4	5+
Current roles in our department	☐	☐	☐	☐	☐	☐
Former roles in our department	☐	☐	☐	☐	☐	☐
Current roles in the institution (outside the department)	☐	☐	☐	☐	☐	☐
Former roles in the institution (outside the department)	☐	☐	☐	☐	☐	☐
Current roles outside the institution	☐	☐	☐	☐	☐	☐
Former roles outside the institution	☐	☐	☐	☐	☐	☐

Academic productivity

13. Have you ever had intramural funding for research or creative work?
    Yes, No
    If yes: number of current and prior intramural grants funded

14. Have you ever had extramural funding for research or creative work?
    Yes, No
    If yes: number of current and prior extramural grants funded

15. Have you ever served on an editorial board or as an Editor for a peer-reviewed publication?
    Yes, No

16. How many of each of the following have you done in the **last 5 years**?

	0	1	2	3	4	5+
Peer-reviewed publications as first, second, or senior author	☐	☐	☐	☐	☐	☐
Departmental awards received	☐	☐	☐	☐	☐	☐
National or institutional awards received	☐	☐	☐	☐	☐	☐
Educational modules, sessions, or lectures developed	☐	☐	☐	☐	☐	☐
Teaching sessions for medical students, residents, or fellows facilitated	☐	☐	☐	☐	☐	☐

Projects related to DEI or wellness completed	☐	☐	☐	☐	☐	☐
Non-promotable activities completed (ex: social event planning, internal committees, internal education)	☐	☐	☐	☐	☐	☐

17. Have you ever been denied an on-time promotion or merit increase?
    Yes, No
    If yes: what was the rationale given?

18. Have you ever received an accelerated promotion or merit increase?
    Yes, No
    If yes: on what basis was the acceleration granted?

19. Do you perceive a difference between academic productivity and career success between women and men in our department?
    Definitely no, probably no, unsure, probably yes, definitely yes

20. Please explain your answer to the above question.

Community and Mentorship

For the purposes of this survey, please use the following definitions:
Ally: A trusted person who supports you and helps you achieve your goals.
Mentor: An advisor who offers advice and/or guidance on career and work, often based on their own experience.
Sponsor: A person who uses their own influence of social capital to create or connect you with career-advancing opportunities.
Impostor Phenomenon: A persistent doubt of one's own abilities and skills (i.e., that your own abilities are not equal to those around you) and fear of being discovered as a "professional fraud"

21. How many of each of the following do you currently have within our department?

	0	1	2	3	4	5+
Allies	☐	☐	☐	☐	☐	☐
Mentors	☐	☐	☐	☐	☐	☐
Sponsors	☐	☐	☐	☐	☐	☐

22. Have you ever participated in a formal coaching program?
    Yes/No
    If yes: What were the benefits of going through a coaching program?

23. To what degree have you experienced impostor phenomenon?
    Not at all, to a minimal extent, to some extent, to a great extent, constantly

24. Except if "not at all" selected: Explain how impostor phenomenon impacts your day-to-day experience.

Intent to stay

25. Are you actively considering leaving our institution?

If yes:
1. What alternative(s) are you considering?
Another academic department, private practice, industry, leave medicine entirely, other (specify)
2. What would be your primary reasons for leaving?

if no:
1. What has influenced your decision to stay?

Well-being

26. How satisfied are you with the following areas of work-life balance?

	Extremely dissatisfied	Somewhat dissatisfied	Neither satisfied or dissatisfied	Somewhat satisfied	Extremely satisfied
Workload	☐	☐	☐	☐	☐
Control	☐	☐	☐	☐	☐
Reward	☐	☐	☐	☐	☐
Community	☐	☐	☐	☐	☐
Fairness	☐	☐	☐	☐	☐
Values	☐	☐	☐	☐	☐

Parenthood

27. Are you a parent? (if no – skip to next section)
If yes:
a. How many children do you have?
b. What are the age(s) of your child(ren)?
c. Are you the sole or primary caregiver for your children?
Yes, No
d. In your household, does one parent take on more responsibility for child-rearing?
Yes, No
If yes: self, other parent
e. Who provides childcare for your child(ren) during work hours?
School, daycare, in-home care provider, other parent, other family member, n/a
f. Who is primarily responsible for transporting your child(ren) to and from school, daycare, or other activities?
Self, partner, other caregiver, n/a
g. How much time per week do you spend arranging caregiving, transportation, activities, or other logistic needs for your child(ren)? (# hours)
h. How many hours per day do you spend with your child(ren) on an average weekday?
i. What are the greatest sources of stress related to balancing your work with parenting?
j. Do you believe that these stressors are different for women and men?
Yes, No
If yes: Explain.

k. What steps could the department take to support parents?
l. What are the greatest sources of work-related stress facing new parents (i.e., children age 2 and under)?
m. What steps could the department take to support new parents (i.e., children age 2 and under)?
n. Have you ever been a lactating parent while working in our department?
   Yes, no
   If yes:
   i. Within the last 5 years?
      Yes, no
   ii. How often did you / do you get dedicated lactation breaks (i.e., from clinical duties) while at work?
      Always (100% of the time), almost always (75-99% of the time), often (50-74% of the time), sometimes (25-49% of the time), rarely (1-24% of the time), never (0%)
   iii. Where did/do you most commonly express breastmilk? For each option, specify the percentage of time:
      Lactation room, office/other private space, shared space (ex: break room, locker room, etc), while working in the OR or another clinical location
   iv. What are/were the most challenging aspects of lactation while working in our department?
   v. What could the department do to support/facilitate lactation for faculty members?
   vi. What is the department already doing to support/ facilitate lactation for faculty members?

28. Are you a caregiver for parents or other family members? (if no – skip to the next section)
    If yes:
    a. What are the greatest sources of stress related to balancing your work with caregiving responsibilities?
    b. What could the department do to support caregivers?

Open-ended questions, reflections, and comments

29. What are the greatest threats to retaining women in our department?
30. What is our department doing to support women faculty members?
31. What are the things you would like to see the department work on to improve the working environment for women?
32. Is there anything else you would like us to know?

*Note: Questions listed in blue are open-ended questions

Appendix 1: Opening Page to Survey

Confidential Survey – Online Information Sheet

We are asking you to take part in a research study being done by [investigator's name] at the [investigator's institution]. Being in this study is optional.

If you choose to be in the study, you will complete a survey. This survey is designed to learn more about your overall experience as a faculty member at [investigator's institution]. The survey will take about 20 minutes to complete. Four survey participants will be chosen at random to receive a $50 gift card as compensation for your time. **You may want to consider having a copy of your CV on hand while completing the survey to more expediently answer questions related to academic productivity**.

You can skip questions that you do not want to answer or stop the survey at any time. The survey is anonymous, and no one will be able to link your answers back to you. Please do not include your name or other information that could be used to identify you in the survey responses.

At the end of the survey, you will be asked to indicate whether you would like to be included in a focus group. If you wish to be included, you will be asked to provide your name, contact information, and some basic demographic information. Please note: the sign-up process for the focus group is not linked to your survey responses and your survey responses will remain anonymous.

Questions? Please contact [investigator] at [investigator's email address]. If you have questions or concerns about your rights as a research participant, you can call the [IRB contact information].

If you want to participate in this study, click the arrow button below to start the survey.

Appendix 2: End of Survey Landing Page

Thank you for participating in our survey.

We would like to invite you to participate in a **focus group** to more deeply understand issues impacting specific groups of **women faculty**: **early career** (i.e., Clinical Instructor or Assistant Professor), **mid-career** (i.e., Associate Professor), **established or late-career** (i.e., Professor), **clinically-focused** (i.e., >50% effort devoted to clinical work), **research-focused** (i.e., >50% effort devoted to research), **parents**, **belonging to an underrepresented group**.

Each focus groups will last approximately **1 hour** and will be scheduled at a mutually convenient time. Participants will be given a **$50 gift card** for their time. If you are interested in participating, **please fill out this form** [hyperlink to form] which will ask for your name, email address, basic demographic information, and focus group preferences. The focus group sign-up form will not be linked to your survey results, so your survey responses will remain anonymous.

If you would like to enter your name into a **drawing for one of four $50 gift cards**, please provide your email address via **this form** [hyperlink to form].Your name will not be associated with your survey responses.
