# Supplementary material for: Understanding professional disparities in academic anesthesiology: a single-center gender-based survey study
Source: BMC Anesthesiol. 2025 Dec 7;26:31. doi: 10.1186/s12871-025-03522-z (PMC12797490; doi:10.1186/s12871-025-03522-z)

Supplemental Document 1 – Table of Contents

Quantitative Survey Results

Supplemental Figure and Table 1: Academic rank by years on faculty
Supplemental Figure and Table 2: Department leadership positions by years on faculty
Supplemental Figure and Table 3: Institutional leadership positions by years on faculty
Supplemental Figure and Table 4: National leadership positions by years on faculty
Supplemental Figure and Table 5: Ever served on an editorial board by years on faculty
Supplemental Figure and Table 6: Number of publications in the past 5 years by years on faculty
Supplemental Table 6.1: 0-2 vs. 3+ publications by years on faculty
Supplemental Figure and Table 7.1 and 7.2: Research funding by years on faculty
Supplemental Figure and Table 8: Number of Departmental Awards by Years on Faculty
Supplemental Figure and Table 9: Number of National Awards by Years on Faculty
Supplemental Figure and Table 10: Non-promotable activity by years on faculty
Supplemental Table 11: Allies, Mentors, and Sponsors
Supplemental Table 12: Reasons for Leaving or Staying
Supplemental Figure 12: Primary parent
Supplemental Table 13: Sources of stress for parents
Supplemental Figure 13: Parenting stress differences between men and women
Supplemental Figure and Table 14: Impostor phenomenon

Qualitative Survey Results

Supplemental Table 15: Representative quotes related to survey themes and sub-themes
Supplemental Figure 15: Potential Interventions to Address Career Advancement Sub-themes
Supplemental Figure 16: Potential Interventions to Address Work Culture Sub-themes
Supplemental Figure 17: Potential Solutions for Providing Balance between Home and Work for Parents

Supplemental Figure 1: Academic Rank by Years on Faculty

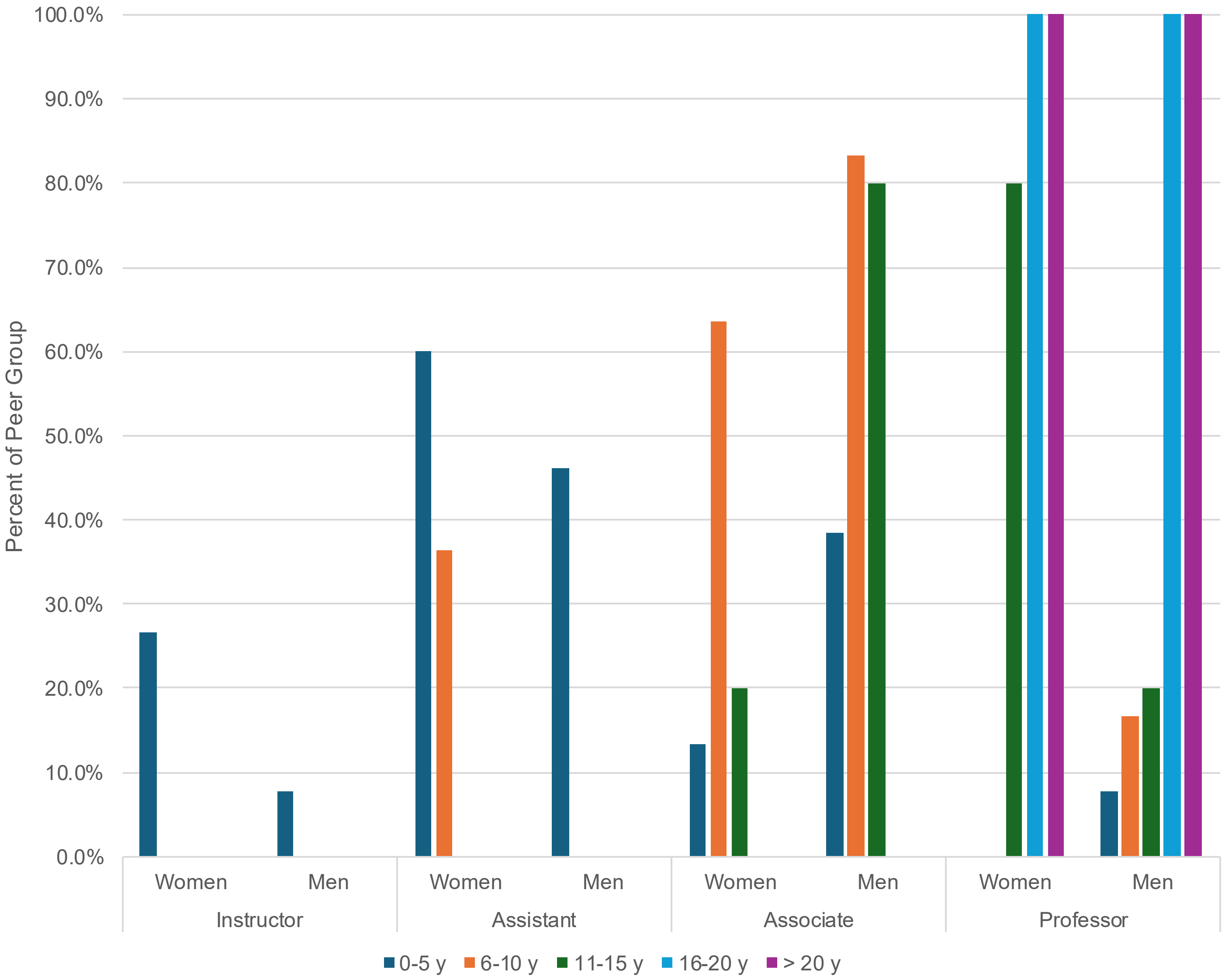

Supplemental Table 1: Academic Rank by Years on Faculty

Duration	Instructor		Assistant		Associate		Professor	
	Women	Men	Women	Men	Women	Men	Women	Men
0-5 y	26.7%	7.7%	60.0%	46.2%	13.3%	38.5%	0.0%	7.7%
6-10 y	0.0%	0.0%	36.4%	0.0%	63.6%	83.3%	0.0%	16.7%
11-15 y	0.0%	0.0%	0.0%	0.0%	20.0%	80.0%	80.0%	20.0%
16-20 y	0.0%	0.0%	0.0%	0.0%	0.0%	0.0%	100.0%	100.0%
> 20 y	0.0%	0.0%	0.0%	0.0%	0.0%	0.0%	100.0%	100.0%

Supplemental Figure 2: Department Leadership Positions by Years on Faculty

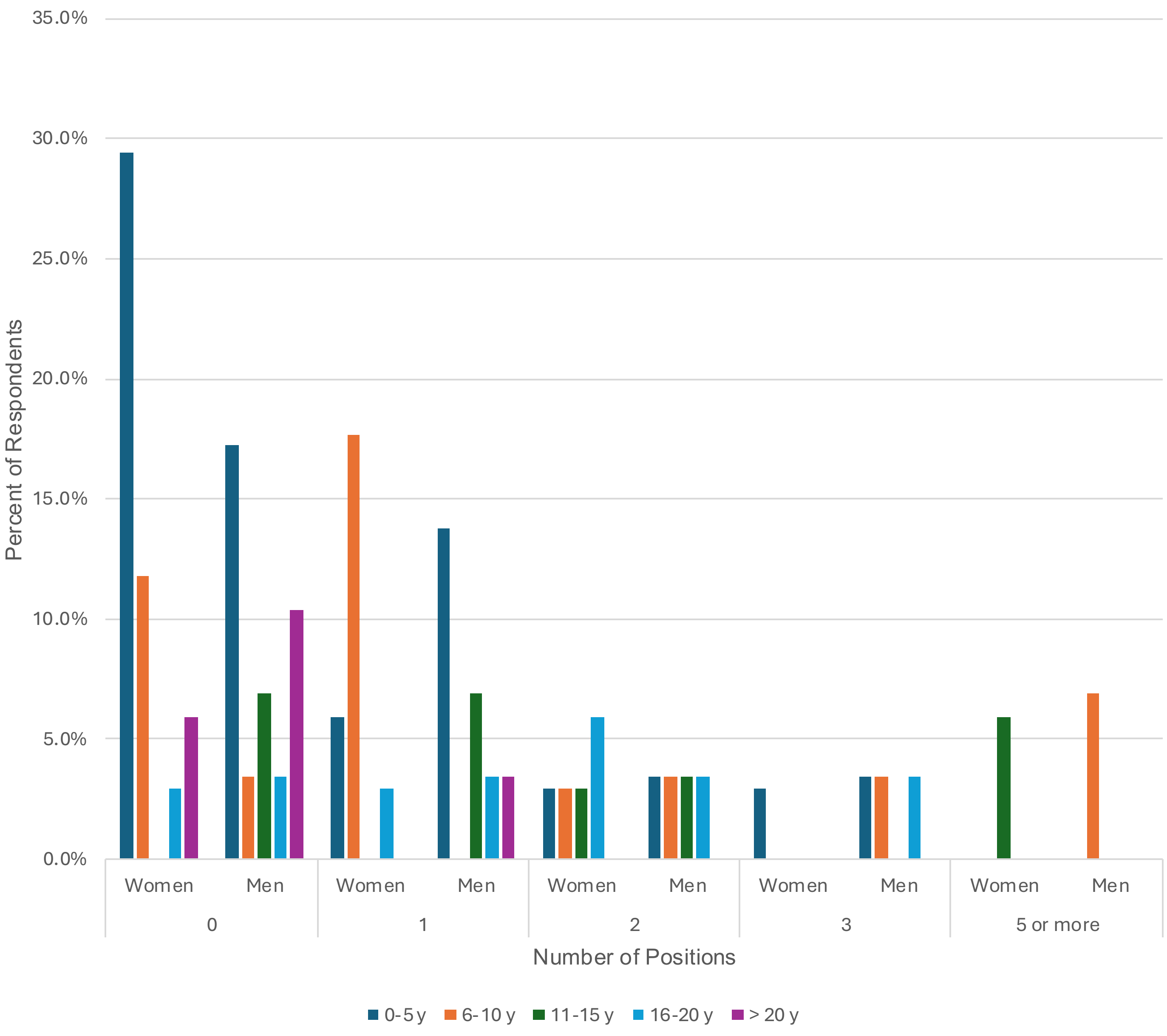

Supplemental Table 2: Department Leadership Positions by Years on Faculty

Duration	0		1		2		3		5 or more	
	Women	Men	Women	Men	Women	Men	Women	Men	Women	Men
0-5 y	29.4%	17.2%	5.9%	13.8%	2.9%	3.4%	2.9%	3.4%	0.0%	0.0%
6-10 y	11.8%	3.4%	17.6%	0.0%	2.9%	3.4%	0.0%	3.4%	0.0%	6.9%
11-15 y	0.0%	6.9%	0.0%	6.9%	2.9%	3.4%	0.0%	0.0%	5.9%	0.0%
16-20 y	2.9%	3.4%	2.9%	3.4%	5.9%	3.4%	0.0%	3.4%	0.0%	0.0%
> 20 y	5.9%	10.3%	0.0%	3.4%	0.0%	0.0%	0.0%	0.0%	0.0%	0.0%

Supplemental Figure 3: Institutional Leadership Positions by Years on Faculty

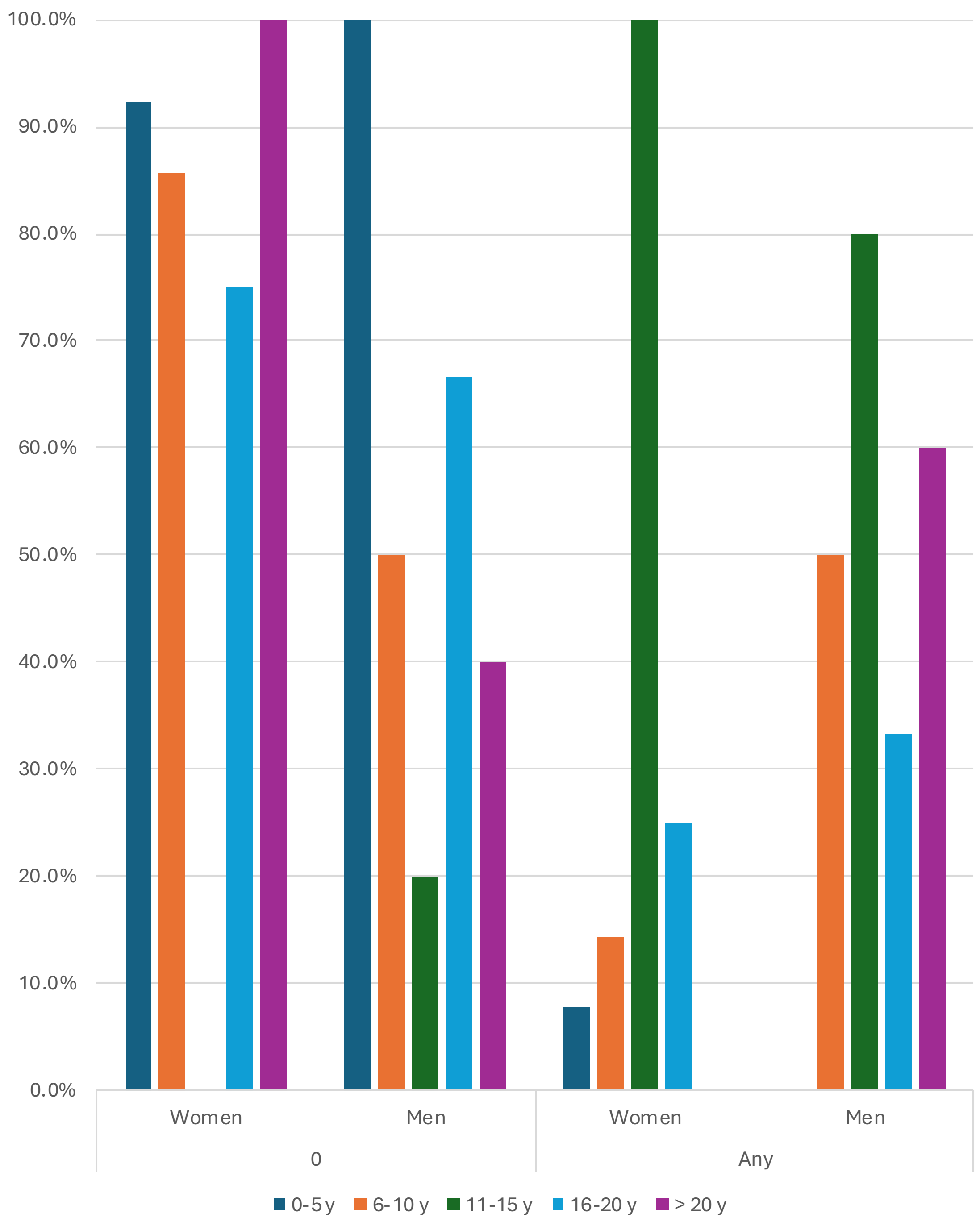

Supplemental Table 3: Institutional Leadership Positions by Years on Faculty

Duration	0		1		2		3		5 or more		Any	
	Women	Men	Women	Men	Women	Men	Women	Men	Women	Men	Women	Men
0-5 y	92.3%	100.0%	0.0%	0.0%	7.7%	0.0%	0.0%	0.0%	0.0%	0.0%	7.7%	0.0%
6-10 y	85.7%	50.0%	0.0%	25.0%	14.3%	0.0%	0.0%	0.0%	0.0%	25.0%	14.3%	50.0%
11-15 y	0.0%	20.0%	0.0%	60.0%	0.0%	0.0%	50.0%	20.0%	50.0%	0.0%	100.0%	80.0%
16-20 y	75.0%	66.7%	25.0%	0.0%	0.0%	0.0%	0.0%	33.3%	0.0%	0.0%	25.0%	33.3%
> 20 y	100.0%	40.0%	0.0%	0.0%	0.0%	20.0%	0.0%	20.0%	0.0%	20.0%	0.0%	60.0%

Supplemental Figure 4: National Leadership Positions by Years on Faculty

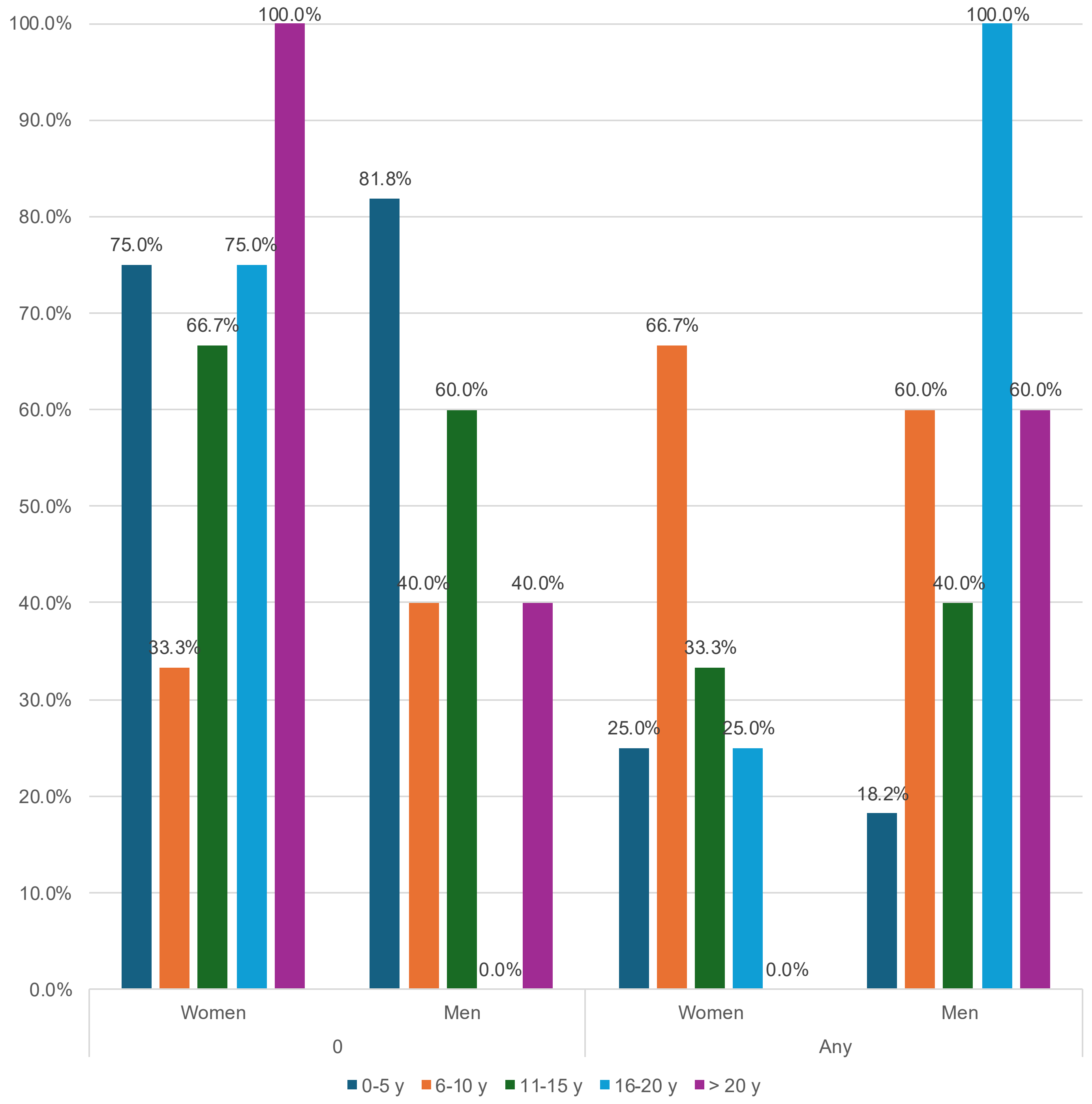

Supplemental Table 4: National Leadership Positions by Years on Faculty

Duration	0		1		2		3		5 or more		Any	
	Women	Men	Women	Men	Women	Men	Women	Men	Women	Men	Women	Men
0-5 y	75.0%	81.8%	16.7%	9.1%	8.3%	9.1%	0.0%	0.0%	0.0%	0.0%	25.0%	18.2%
6-10 y	33.3%	40.0%	50.0%	40.0%	0.0%	0.0%	16.7%	0.0%	0.0%	20.0%	66.7%	60.0%
11-15 y	66.7%	60.0%	0.0%	20.0%	0.0%	20.0%	0.0%	0.0%	33.3%	0.0%	33.3%	40.0%
16-20 y	75.0%	0.0%	0.0%	100.0%	0.0%	0.0%	25.0%	0.0%	0.0%	0.0%	25.0%	100.0%
> 20 y	100.0%	40.0%	0.0%	40.0%	0.0%	0.0%	0.0%	20.0%	0.0%	0.0%	0.0%	60.0%

Supplemental Figure 5: Ever Served on an Editorial Board by Years on Faculty

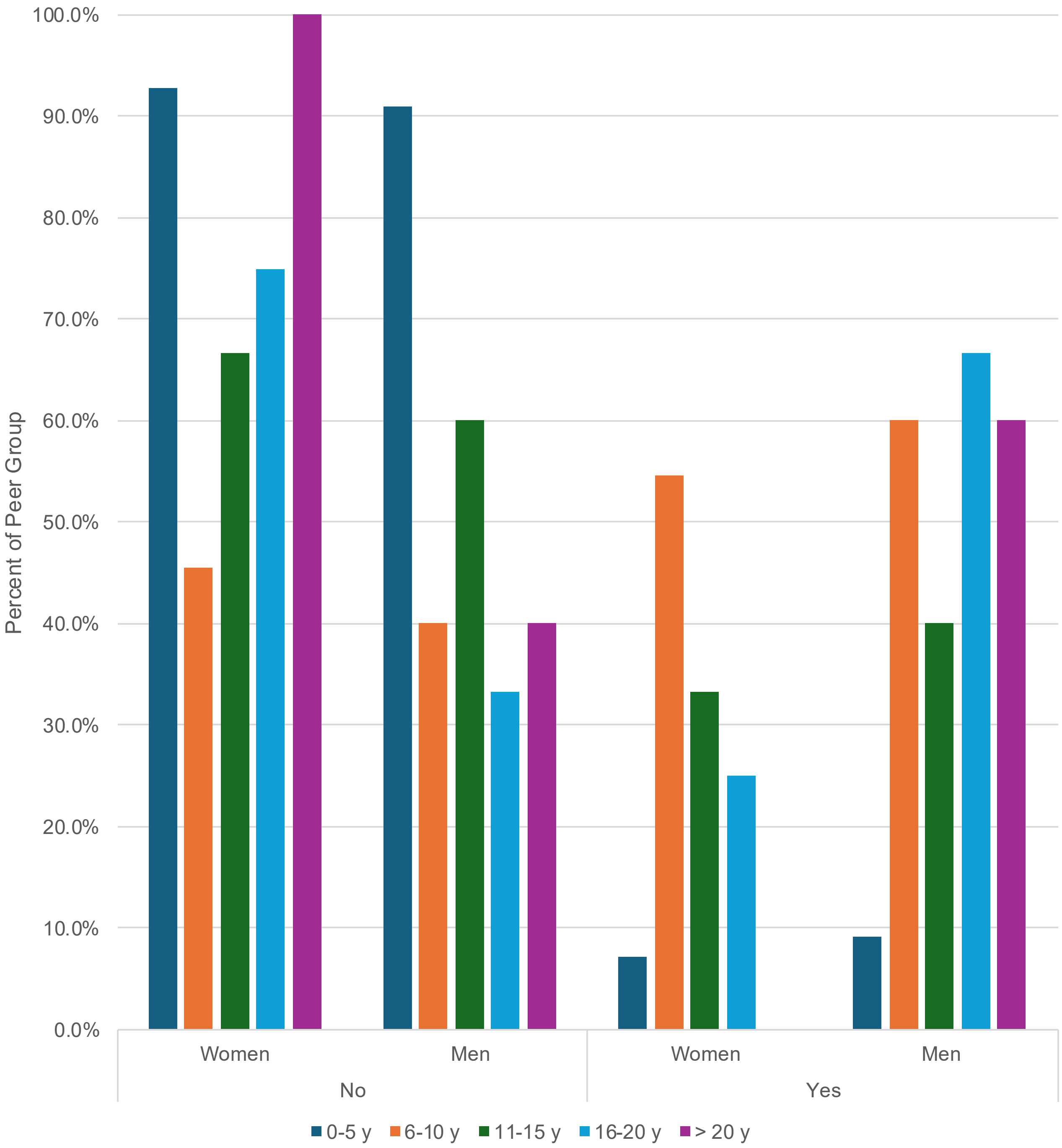

Supplemental Table 5: Ever Served on Editorial Board by Years on Faculty

Duration	No		Yes	
	Women	Men	Women	Men
0-5 y	92.9%	90.9%	7.1%	9.1%
6-10 y	45.5%	40.0%	54.5%	60.0%
11-15 y	66.7%	60.0%	33.3%	40.0%
16-20 y	75.0%	33.3%	25.0%	66.7%
> 20 y	100.0%	40.0%	0.0%	60.0%

Supplemental Figure 6: Number of Publications (Past 5 Years) by Years on Faculty

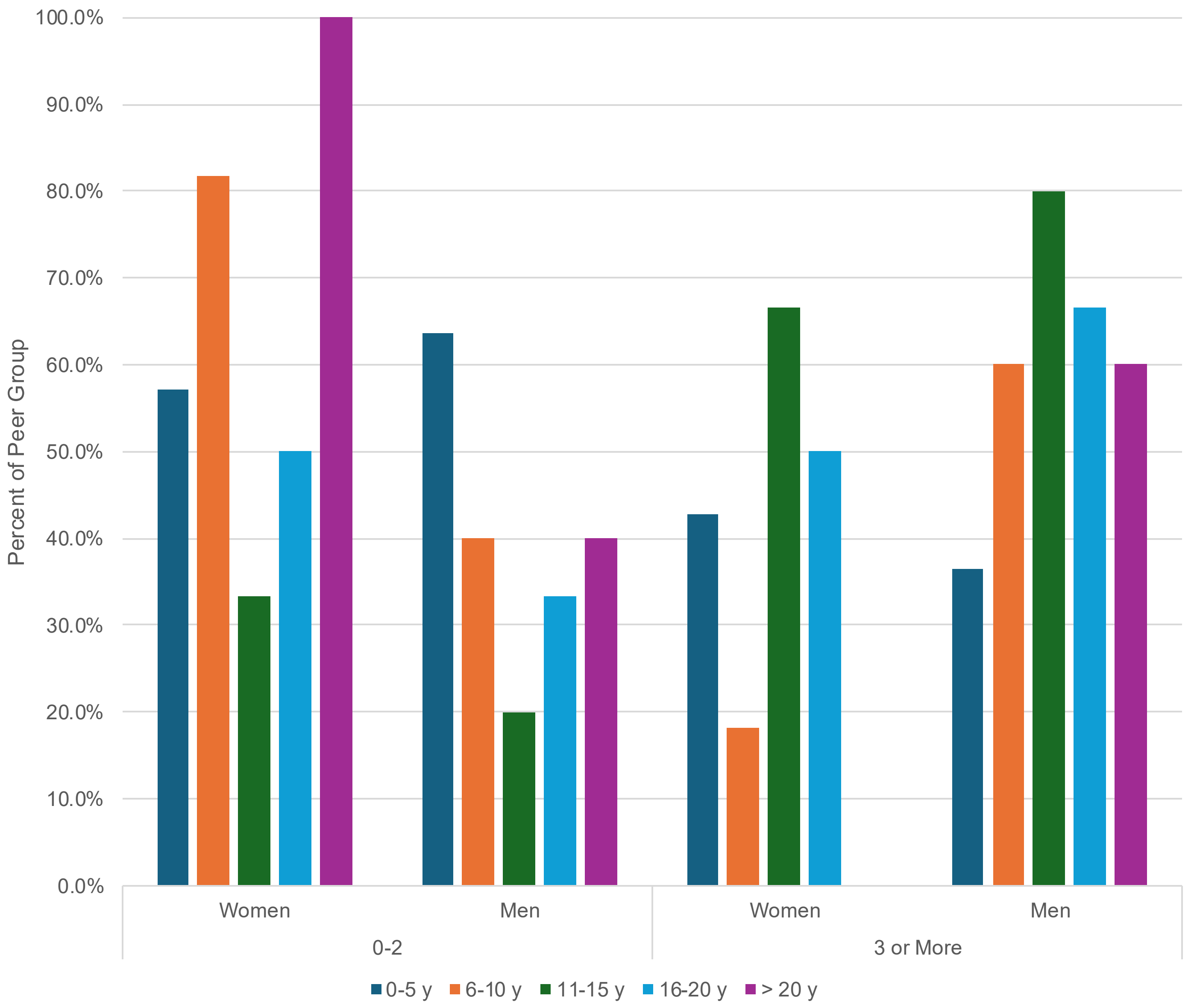

Supplemental Table 6.1: Number of Publications by Years on Faculty

Duration	0		1		2		3		4		5 or more	
	Women	Men	Women	Men	Women	Men	Women	Men	Women	Men	Women	Men
0-5 y	28.6%	36.4%	21.4%	9.1%	7.1%	18.2%	7.1%	9.1%	7.1%	0.0%	28.6%	27.3%
6-10 y	27.3%	20.0%	54.5%	20.0%	0.0%	0.0%	0.0%	20.0%	0.0%	20.0%	18.2%	20.0%
11-15 y	33.3%	20.0%	0.0%	0.0%	0.0%	0.0%	0.0%	20.0%	0.0%	0.0%	66.7%	60.0%
16-20 y	0.0%	0.0%	25.0%	33.3%	25.0%	0.0%	25.0%	0.0%	0.0%	0.0%	25.0%	66.7%
> 20 y	0.0%	20.0%	50.0%	20.0%	50.0%	0.0%	0.0%	20.0%	0.0%	20.0%	0.0%	20.0%

Supplemental Table 6.2: 0-2 vs. 3+ Publications by Years on Faculty

Duration	0-2		3 or More	
	Women	Men	Women	Men
0-5 y	57.1%	63.6%	42.9%	36.4%
6-10 y	81.8%	40.0%	18.2%	60.0%
11-15 y	33.3%	20.0%	66.7%	80.0%
16-20 y	50.0%	33.3%	50.0%	66.7%
> 20 y	100.0%	40.0%	0.0%	60.0%

Supplemental Figure 7.1: Ever Received Extramural Research Funding by Years on Faculty

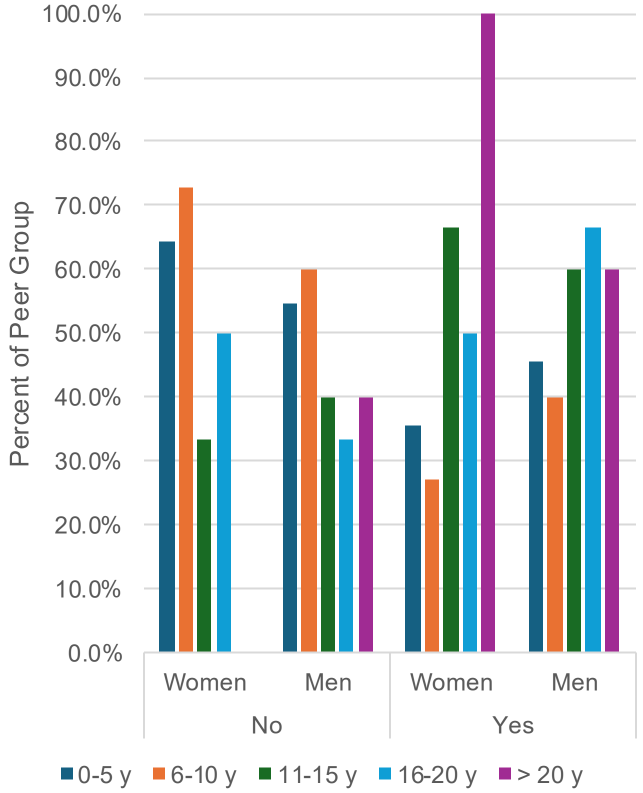

Supplemental Table 7.1: Ever Received Extramural Funding by Years on Faculty

	No		Yes	
Duration	Women	Men	Women	Men
0-5 y	64.3%	54.5%	35.7%	45.5%
6-10 y	72.7%	60.0%	27.3%	40.0%
11-15 y	33.3%	40.0%	66.7%	60.0%
16-20 y	50.0%	33.3%	50.0%	66.7%
> 20 y	0.0%	40.0%	100.0%	60.0%

Supplemental Figure 7.2: Ever Received Intramural Research Funding by Years on Faculty

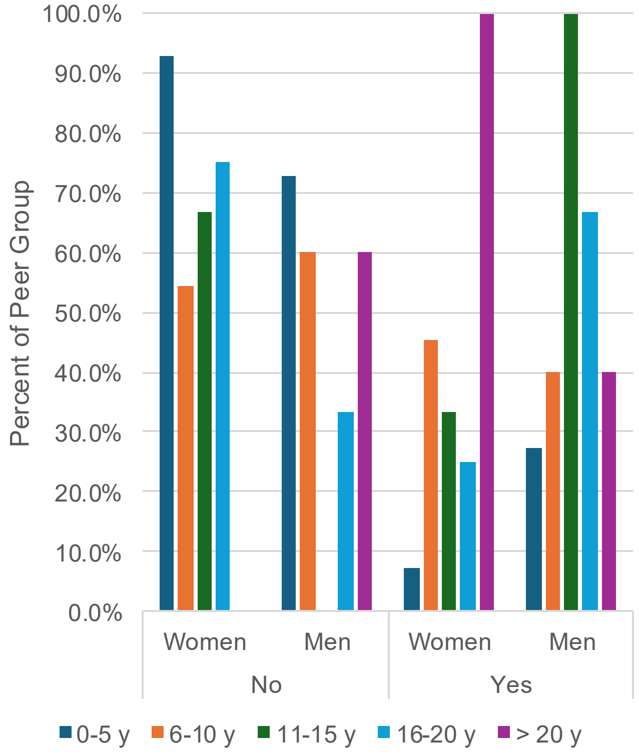

Supplemental Table 7.2: Ever Received Extramural Funding by Years on Faculty

	No		Yes	
Duration	Women	Men	Women	Men
0-5 y	92.9%	72.7%	7.1%	27.3%
6-10 y	54.5%	60.0%	45.5%	40.0%
11-15 y	66.7%	0.0%	33.3%	100.0%
16-20 y	75.0%	33.3%	25.0%	66.7%
> 20 y	0.0%	60.0%	100.0%	40.0%

Supplemental Figure 8: Number of Departmental Awards (Past 5 Years) by Years on Faculty

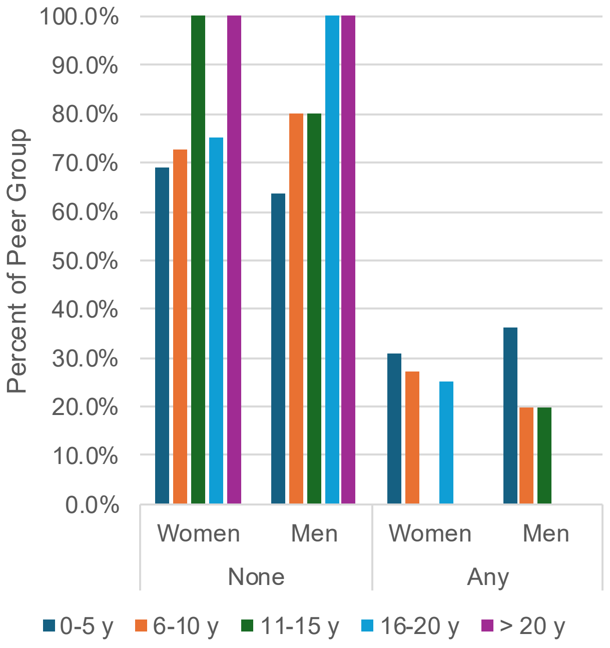

Supplemental Table 8: Number of Departmental Awards by Years on Faculty

Duration	None		Any	
	Women	Men	Women	Men
0-5 y	69.2%	63.6%	30.8%	36.4%
6-10 y	72.7%	80.0%	27.3%	20.0%
11-15 y	100.0%	80.0%	0.0%	20.0%
16-20 y	75.0%	100.0%	25.0%	0.0%
> 20 y	100.0%	100.0%	0.0%	0.0%

Supplemental Figure 9: Number of National Awards (Past 5 Years) by Years on Faculty

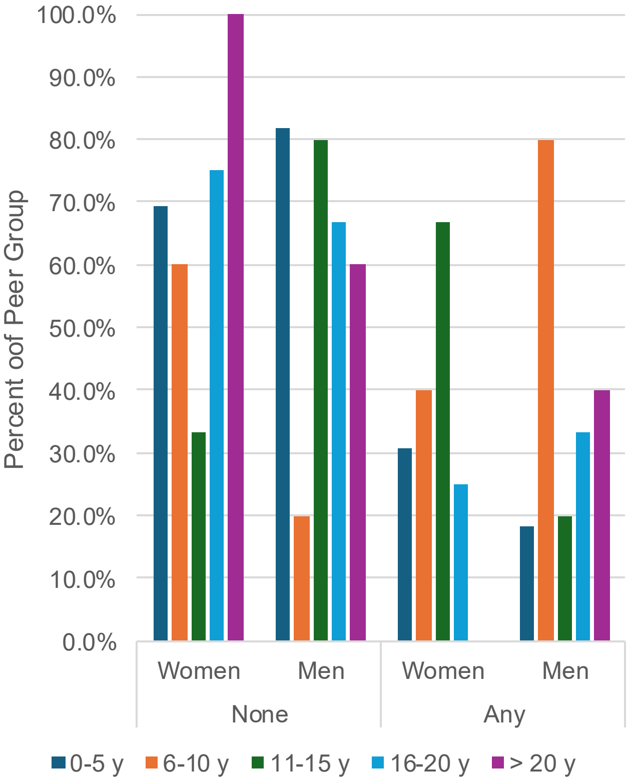

Supplemental Table 9: Number of National Awards by Years on Faculty

Duration	None		Any	
	Women	Men	Women	Men
0-5 y	69.2%	81.8%	30.8%	18.2%
6-10 y	60.0%	20.0%	40.0%	80.0%
11-15 y	33.3%	80.0%	66.7%	20.0%
16-20 y	75.0%	66.7%	25.0%	33.3%
> 20 y	100.0%	60.0%	0.0%	40.0%

Supplemental Figure 10: Non-promotable Activities by Years on Faculty

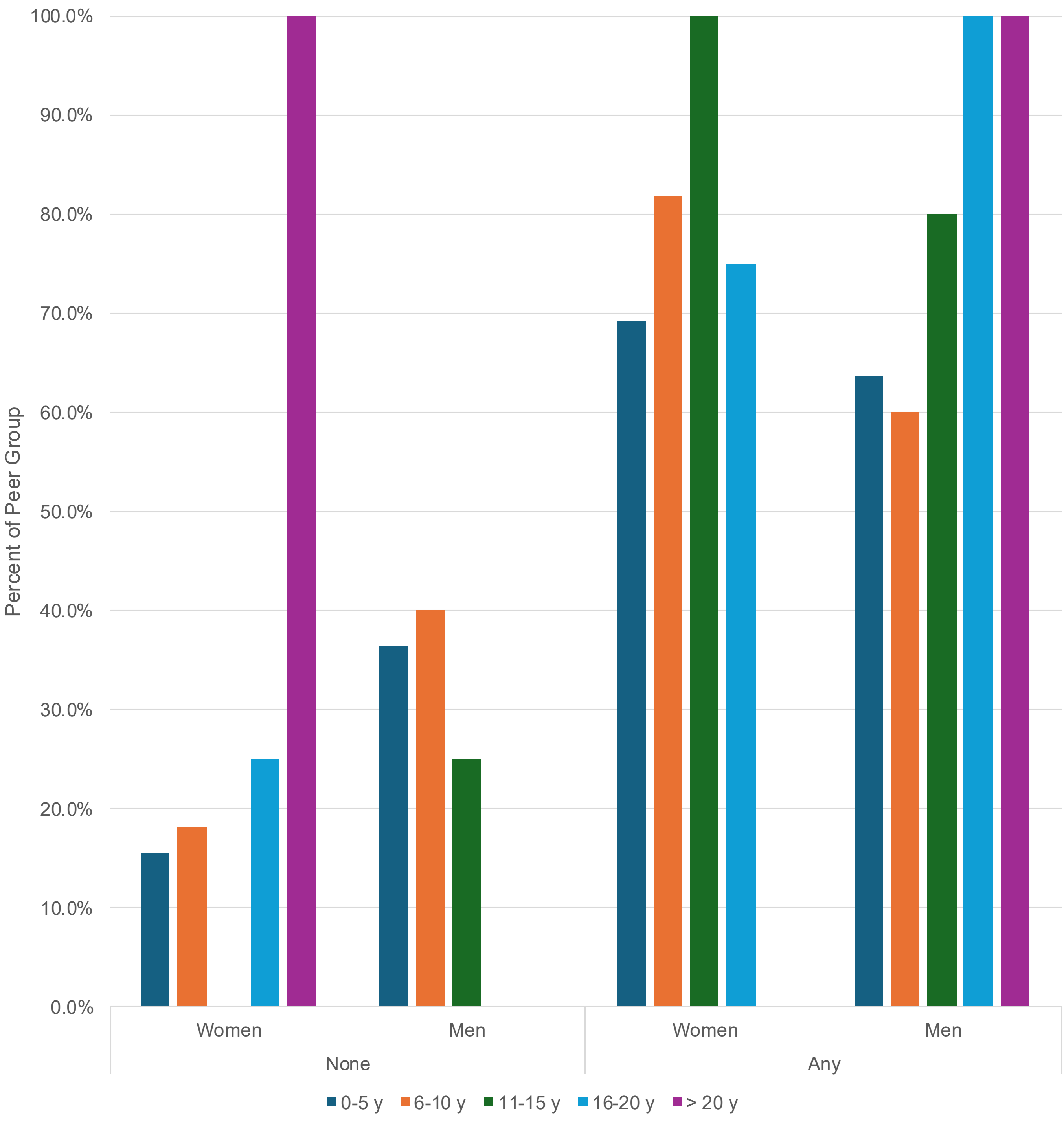

Supplemental Table 10: Non-Promotable Activities by Years on Faculty

Duration	None		Any	
	Women	Men	Women	Men
0-5 y	15.4%	36.4%	69.2%	63.6%
6-10 y	18.2%	40.0%	81.8%	60.0%
11-15 y	0.0%	25.0%	100.0%	80.0%
16-20 y	25.0%	0.0%	75.0%	100.0%
> 20 y	100.0%	0.0%	0.0%	100.0%

Supplemental Table 11: Allies, Mentors, Sponsors

Number	Allies		Mentors		Sponsors	
	Women	Men	Women	Men	Women	Men
0	15.6%	22.2%	28.1%	42.3%	41.9%	57.7%
1	25.0%	7.4%	34.4%	19.2%	38.7%	26.9%
2	15.6%	22.2%	28.1%	26.9%	16.1%	15.4%
3	6.3%	7.4%	6.3%	3.8%	3.2%	--
4 or more	37.5%	40.7%	3.1%	7.7%	--	--
Any	84.4%	77.8%	71.9%	65.4%	58.1%	42.3%

Supplemental Table 12: Reasons to Leave and Stay

Potential reasons for leaving	
Category	***Exemplar quotes***
Work-life integration	" While I enjoy my role as an anesthesiologist, balancing parenting (despite having childcare support) with long hours is challenging. Limited time with my children affects our connection and my ability to support them." (F)<br>"Work life balance remuneration" (F)
Clinical schedule	"Little predictability in depart times particularly at [one clinical location]." (F)
Work culture	"Lack of a good work culture…between periop teams. [Being asked to do] menial tasks, long turnover times, Poor compensation for [my] subspecialty…exorbitant parking and dilapidated elevators with little help moving patients etc." (F)
Career advancement	"Unable to achieve any independence, development of my directorship abilities, or gain any type of respect…" (F)<br>"Opportunity for leadership" (F)
Academic medicine	"The academic work environment's bureaucracy adds to this strain. Pursuing projects to enhance patient care faces obstacles, making private practice with flexible hours and higher pay appealing." (F)<br>"there is no transparence in academic institutions about respective individual benefit" (M)
Other opportunities	"Creative endeavors, self actualization, and the fact that I am over 50 and could afford to retire." (M)<br>"Return to training in home country" (M)
**Reasons for staying**	
Carer goals	"I think my goals, and the goals the department has for me, are very well aligned."<br>"Early career, still hoping for a positive trajectory"
People	"Great place to work. Caring team"<br>"I really like the people"<br>"We have a friendly environment and I love the people I work with."<br>"I have received significant support from peers and would not want to let them down." (M)<br>"The people - I work in a group that is very supportive of one another and have several very close friends here." (M)
Academic mission	"My mentors, career advancement…my colleagues, the hospital, the community, and the mission." (W)<br>"Commitment to current work and residents" (W)<br>"I'm appreciative of my opportunities for research, education and clinical care." (M)<br>"The people, academic excellence, work." (M)
Personal/professional fulfillment	"I think my work here is meaningful and I enjoy doing it." (W)<br>"happy in position i am currently in" (M)<br>"I like the work I do." (M)<br>"I enjoy my work." (M)<br>"I'm content." (M)<br>"I like the work I get to do at UCSF, and it is not clear to me that I would be able to be part of such a good subdivision elsewhere." (M)
Infrastructure / Support	"The department values my contributions as a researcher (in terms of salary and with protected time) and supported me when I was going through a difficult time in my personal life."<br>"My clinical colleagues are also incredibly supportive and they allow me to rely on their clinical expertise when needed. They have never made me feel less-than for not having as much clinical experience as they do."<br>"The department has been incredibly supportive of me through my pregnancies and injuries, I'm very grateful."<br>"The research community and resources are outstanding."<br>"Current support from leadership…in pursuing academic interests while maintaining good work/life balance." (M)
Location / Family	"Family, San Francisco, Friends"<br>"Location is close to family"<br>"Family stability. Kids are going to school. Like to give them a steady environment."<br>"Also, my spouse's job limits me to just a couple of cities in the US and the alternatives in those cities are not attractive." (M)<br>"Also, family happy here, I like kayaking with sea otters, etc." (M)

Supplemental Figure 12: Are you the Primary Parent in Your Household?

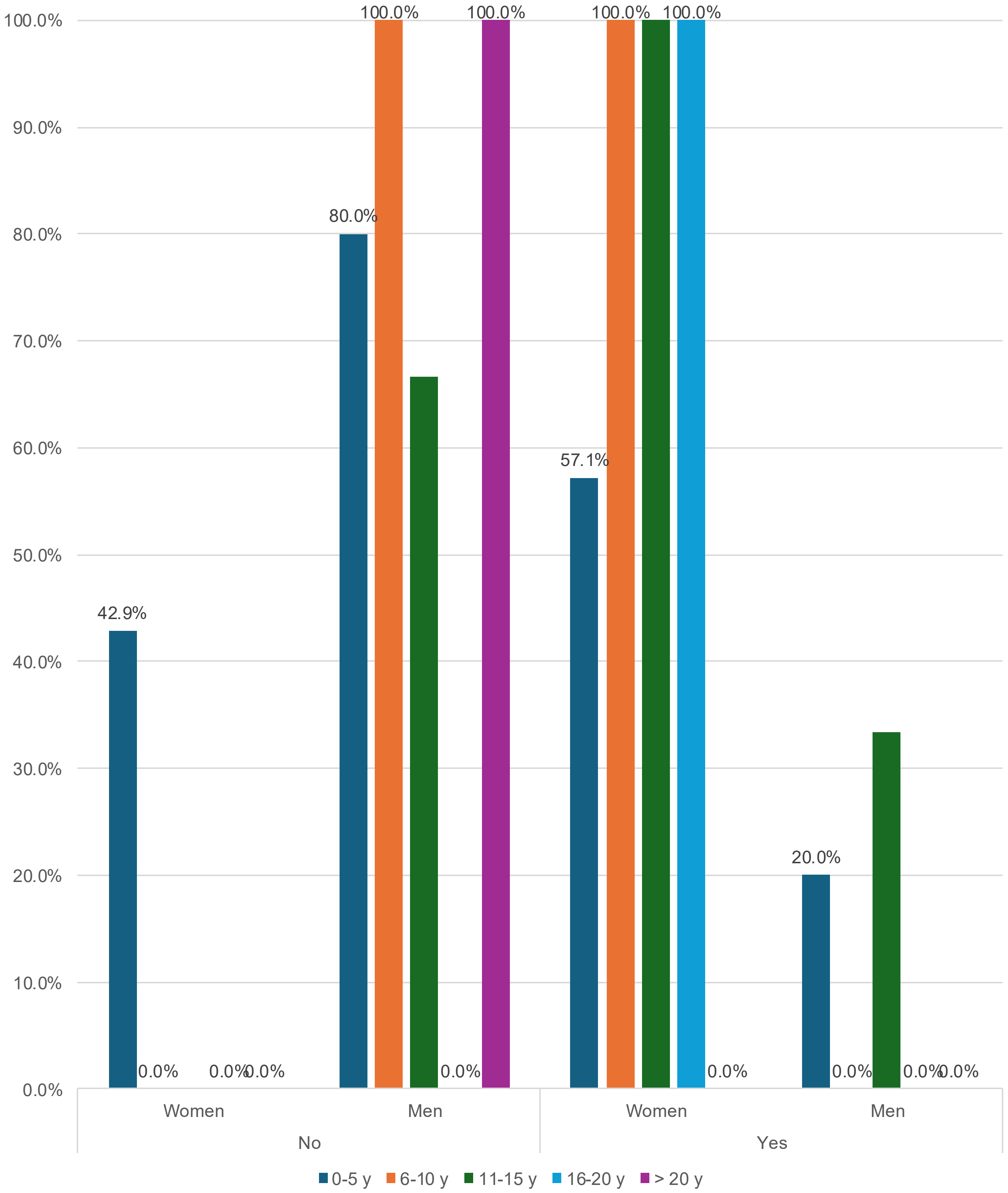

Supplemental Table 13: Sources of stress for parents

Theme	Exemplar Quotes
Cost and Availability of Childcare	Childcare is very difficult to find for infants in [our city], most folks have to find nannies until they get off a waitlist to a daycare somewhere, including the [local] daycare. Being faculty doesn't guarantee that you get off the waitlist in a timely fashion. You literally have to sign up when you're still pregnant to have any hope of getting off the [local] childcare waitlist when you need it. (F/Assoc)
	More resources for childcare--more options AND more importantly, increased pay (could be in the form of stipends, etc). (F/Assoc)
	Reliable, affordable child care. (F/Asst)
	Childcare. (F/Asst)
	On-site daycare that is actually accessible. (F/Asst)
	Increased salary and increased resources for childcare. (F/Assoc)
	Provide onsite childcare to works with anesthesia work hours. (F/Asst)
	It used to be covering childcare when she was younger. (M/Assoc)
	Provide easier access for childcare. Subsidize childcare. (F/Assoc)
Desire for Presence at Home	Women often carry greater guilt when not present for their children. (F/Assoc)
	not seeing my kids (F/Assoc)
	being available for activities when needed (F/Prof)
	I feel that I'm shortchanging my children because I have to work so much (including often on weekends). (F/Prof)
	Having quality time with them independent of the work that everyone needs to do. (M/Assoc)
	desire to be present (M/Prof)
	The fact that I cannot see them before they go to school, I cannot commit to a lot of the school activities, I can only occasionally participate in activities at school and very often I do not have enough energy left in the evening to have a meaningful and fun interaction with them. It takes deliberate effort on my part to be able to do that. (F/Prof)
Flexibility and Predictability	Variable scheduling, lack of control over which days one is working. (F/Prof)
	flexibility for shifts, better more reliable backup (guaranteed drop in care for well and sick children) if we are expected to come to work. (F/Assoc)
	More schedule flexibility and predictability. Sick days! (F/Assoc)
	Allow more flexibility in the timing of shifts, schedules. Either earlier leave times or later leave times. Allow for partial shifts. (F/Assoc)
	not knowing when I will end my day, whether I can make it to afternoon activities or pickup (F/Assoc)
	A few negatives are inflexible scheduling..., daily time unpredictability, and no location preference for working sites. If I could work at the locations closer to my house I might be able to spend more time with my kids, I waste almost 1.5-2 hours in commute due to traffic vs 20-45 minutes. This is purely based on the proximity of certain sites. (F/Assoc)
	Minimize variability in end time so that new parents can reliably be home for dinner/bedtime. (F/Prof)
	The unpredictability of our schedule from day to day week to week and month to month make it IMPOSSIBLE to reliably bring kids to activities, practices, games, swimming, etc. (F/Prof)

Supplemental Table 13 (cont): Sources of stress for parents

Ability to Contribute	Misalignment of daycare/school hours with clinical responsibilities is a humongous problem (less so for a mostly-researcher); literally could not contribute to drop-off or pickup on 80% of clinical days, which forces an imbalance in responsibilities that women "pay for" in other ways. (F/Asst)
	This is also stressful because we are rarely the parents who offer carpools/rides, etc given the unpredictable timing of when we finish in the OR, etc. This has required outsourcing and also means we often miss important events in our children's lives. (F/Prof)
	school, driving kids to activities (M/Asst)
	To get quality time with them. Organizing all the food they get over the day if you want to do it in a healthy way. (F/Asst)
Lactation	As an attending, you just CAN'T be "unavailable", and it's cumbersome to have someone cover for you. It's even more magnified with E1PM when there is literally nobody else to cover for you. The ORs are so unpredictable that it's impossible to be certain when you could realistically take your next pump break, and you end up compromising on this; "I'll do it after induction" has a different meaning when you know +/-15 minutes the induction time, versus +/-2 hours. There are also a lot of variabilities among lactating women: some have no concerns about supply, some really MUST pump every 4 hours or lose supply; some take 10 minutes (I was like that, luckily) and some need 30 minutes. (F/Assoc)
	comments from colleagues, people not understanding that lactation is not a break, trying to eat/hydrate and lactate in the allotted time. Make it clear that lactation is not a break, make OR assignments more manageable (F/Asst)
	E1 can offer to cover rooms during lactation (F/Asst)
	If moms are still nursing/pumping breastmilk, having someone designated to get those moms out on time, especially if they're residents or solo faculty, and not making them feel bad about asking for those breaks. I think also creating some sort of exchange so folks can pass their pumping gear to other colleagues when they're finished pumping might be helpful. I think there is a parent and pets slack channel but it might be too public for folks to feel comfortable posting there. (F/Prof)
	In order to not feel like a burden on my clinical colleagues, I would do double duty during all my lactation breaks, so I would try to eat, pee, and pump within 20-30 minutes, but that meant I had to pack my lunch and 2 snacks every time I was working in the OR, and every second of the break was on the clock. I found that very stressful. I also had to bring all my own pumping gear to and from work. There are small portable pumps now which make it much easier but not everyone has access to those. (F/Prof)
	There is no dedicated break in our line of work. We just choose when to pump based on clinical work which is frustrating. Instead of getting coffee or lunch, I would pump which was exhausting and depressing. (F/Assoc)

Supplemental Figure 13: Are Parenting Stressors Different for Men and Women?

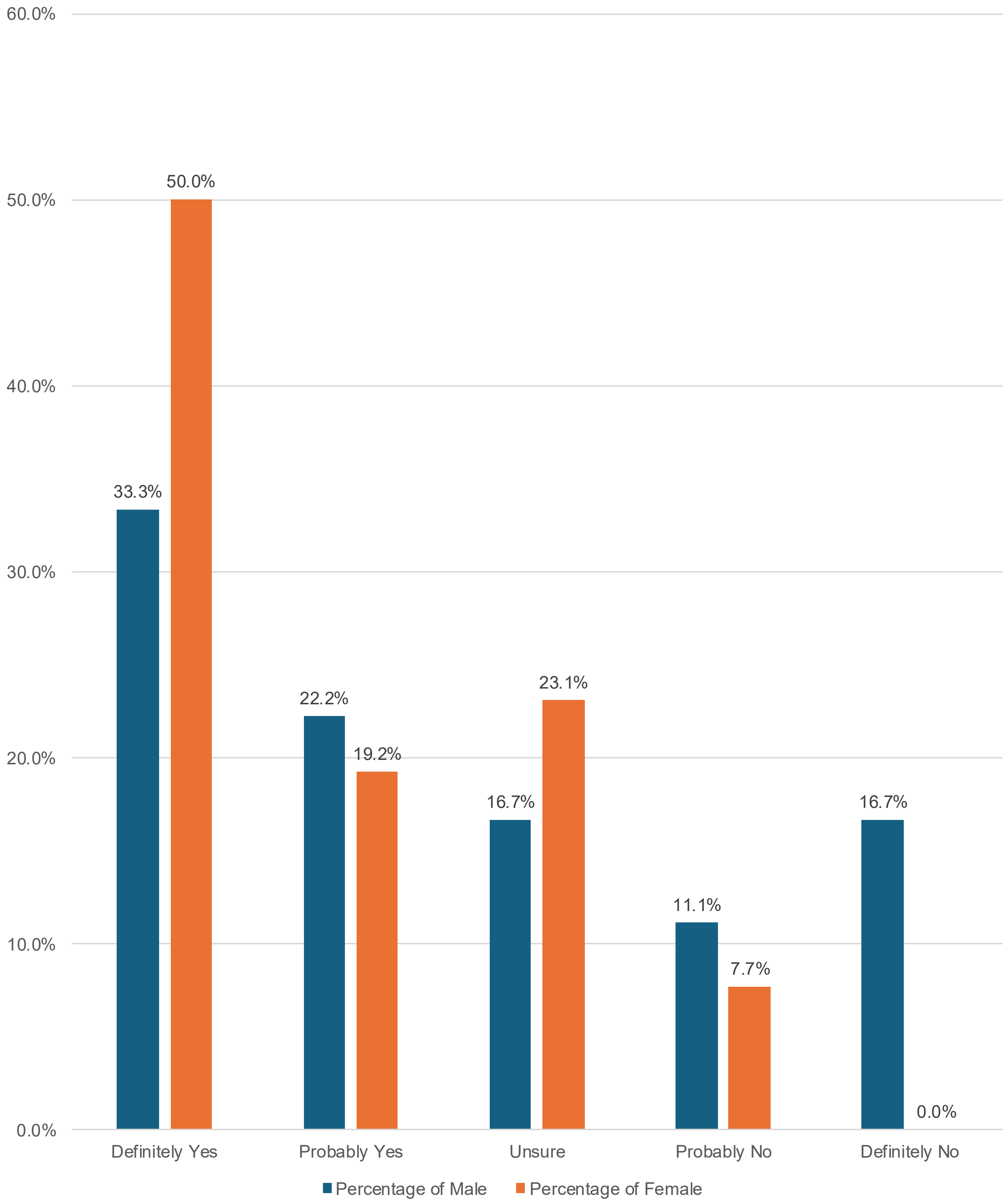

Supplemental Figure 14: To What Extent Do You Experience Impostor Phenomenon?

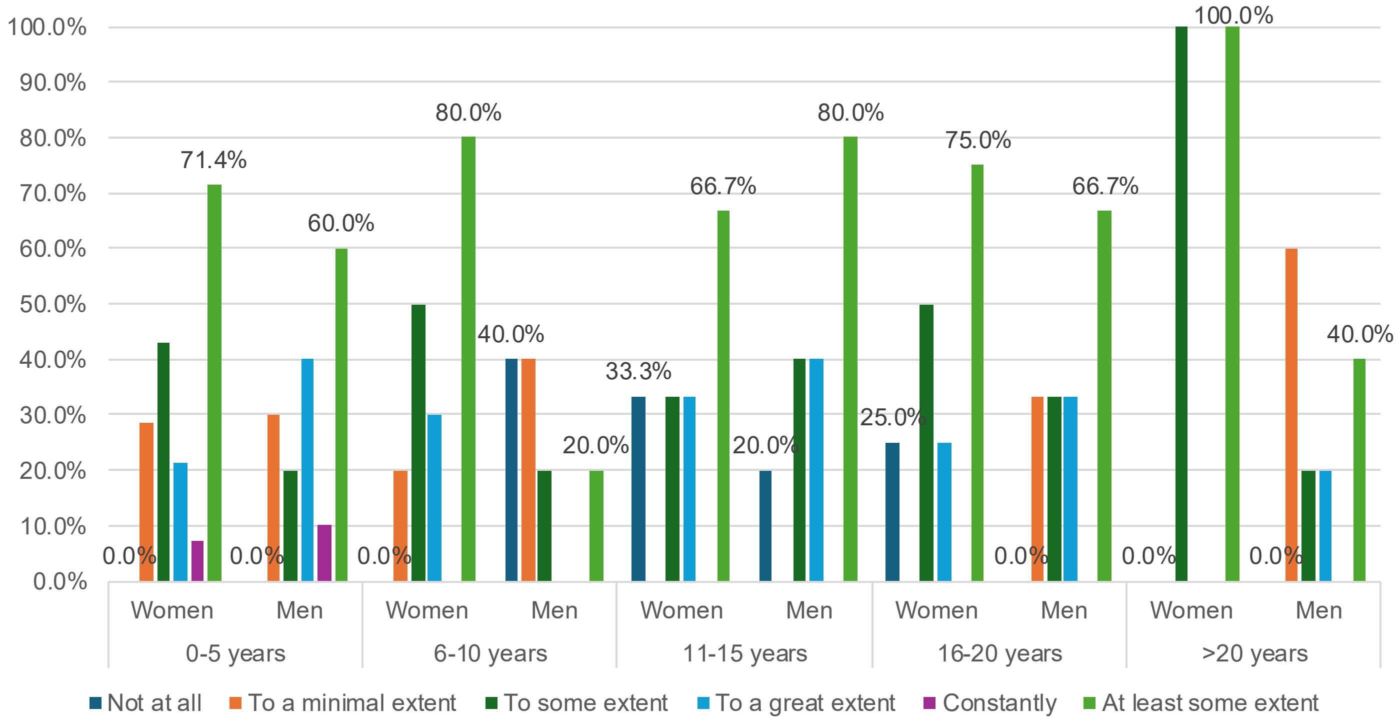

Supplemental Table 14: "Explain how impostor phenomenon impacts your day-to-day work experience."

Gender	Rank	Comments
Woman	Asst	I am very conscious of my abilities and limitations, and probably do not lead with the same degree of confidence as others despite having similar skill sets
Woman	Asst	It creates unhealthy work atmosphere and affects clinical performance
Woman	Asst	I experience it a lot less than I used to. During early career, it was quite substantial, but now I have more concrete evidence of my contributions to my field, which helps.
Woman	Asst	Impacted significantly during training period
Woman	Asst	Work harder
Woman	Asst	It is a part of being a minority woman physician in medicine. It has dissipated over the years but is actively fed when acts of micro and macro aggression present themselves.
Woman	Assoc	Despite being an accomplished researcher, I do not feel like I actually know the stuff that people think I know within the research domain. Admittedly, I probably do know a lot more about research than my non-research colleagues, but when I am among a cohort of my research peers or superiors, I frequently feel like I don't belong.
Woman	Assoc	Second guess decisions
Woman	Assoc	I'm not sure that affects my day to day, but it does affect how aggressively I pursue opportunities whether leadership, research, or educational.
Woman	Assoc	I am very conscious of my abilities and limitations, and probably do not lead with the same degree of confidence as others despite having similar skill sets
Woman	Assoc	It creates unhealthy work atmosphere and affects clinical performance
Woman	Prof	Always questioning the why Me
Woman	Prof	It doesn't. It mostly comes up when I participate in academic pursuits
Woman	Prof	Sometimes makes me feel like I'm not actually successful - like I'm playing the game of showing that I know what I'm doing. Also, there are times that I am asked to intervene or mediate situations that I don't feel properly trained to handle. Then I look around, and see that no one else seems to be trained to handle these situations.
Woman	Prof	This does not impact me day to day. It impacts me in terms of how I see myself within the broader UCSF community. Other departments have a lower overall FTE so people have time to focus heavily on academic work/productivity. We are clinically driven with a higher FTE so it's harder to be productive in terms of academic work.
Man	Asst	Sometimes feel lesser than more senior faculty
Man	Asst	I sometimes think I'm not knowledgeable enough to match expectations.
Man	Asst	not my day to day work
Man	Asst	it can be a distraction, decrease my motivation, kill momentum for working on projects, or prevents me from asking for help
Man	Asst	Causes stress when conducting clinical and non-clinical activities
Man	Assoc	I doubt myself and tend to judge myself a bit too harshly on errors
Man	Assoc	Overall I have learned to deal with it, as objectively with over 20 years of experience and good outcomes/satisfied patients and getting lots of patients out of sticky situations, the logical part of my brain tells me I am not an impostor, and in the process of patient care itself, I am able to turn it off enough to do what is needed. It is useful to keep me from being arrogant and keep me talking out loud, as thankfully I am not scared or concerned at what others may think of me.
Man	Assoc	Just feels more stressful and pressure to perform up to standard
Man	Assoc	I try to assume that others experience impostor phenomenon too, and ignore it.
Man	Assoc	Imposter syndrome (so I read) may not actually be negative - it may spur us to strive to do better and not be overly confident.. imagine someone without any imposter syndrome... that sounds like an overconfident, unaware individual.
Man	Prof	it doesnt
Man	Prof	None
Man	Prof	I had a great dean for student affairs in medical school who gave us an in-depth lectureabout this topic (she was a psychiatrist by training). This has made me aware of it since early in medical school where I felt like a complete impostor early on (I got into my top choice school off the wait list at the last minute - a major contributor). I have had insight into this which has helped (as has the duration and trajectory of my career) but I tend to seek validation from others about tough decisions and generally feel uncomfortable moving forward on some initiatives if I don't feel there is broad consensus. I have tried hard to work on strengthening a core set of values to help me navigate these situations but the doubt is always in the back of my mind.

Supplemental Table 15: Representative quotes related to survey themes and sub-themes

Theme	Sub-Theme	Exemplar Quotes
Career Advancement	Mentorship and Sponsorship	Provide more leadership roles for women especially those in early career stage, provide formal mentorship and sponsorship opportunities. (F/Asst)<br><br>Assign each and every one a sponsor and mentor to define and understand what their true gifts are and cultivate their skills. We are all physicians that are in the top 1% of this country. We all have a lot of skill and a department that can raise each and every one of us. Would only strive to be a standard model for all other universities to follow. (F/Prof)<br><br>… I could envision a women-specific coaching/mentoring system with the focus of success/advancement… (M/Asst)<br><br>Continue work to create opportunities for mentorship and sponsorship and, importantly, grow the capacity for the men to be allies. (M/Prof)
	Promotion Pathways for Women	Women in leadership positions of power, not just DEI positions. More women faculty with designations of Associate Professor and Professor. (F/Asst)<br><br>I feel like there have been many talented women in our department who have not had the same early career opportunities as their male colleagues. Some of these women are still working in our department in mostly clinical roles, while others have left the institution within their first 5 years on faculty. (F/Assoc)<br><br>Create a formal leadership pipeline/retention plan for women faculty. (F/Assoc)<br><br>Lack of upward movement, lack of women in leadership (F/Assoc)<br><br>There is a predominance of white men in leadership positions (F/Assoc)<br><br>… most opportunities are not designed for working mothers- e.g. I cannot be on call all night for an administrative leadership position when I'm also taking care of an infant all night as needed. If there was a hard time cut off or an assistant to help, it might be easier to organize and tackle. But the way things are set up you always have to choose and for most working moms, family is the priority. (F/Assoc)<br><br>I suspect that there could be discrepancy in acceleration rate, because that involves putting one's name forward, which data suggest men are willing to do with much less justification than women! I [previously] had to be willing to ignore [unhelpful] advice and push myself forward as worthy of acceleration. (M/Assoc)<br><br>I do perceive that my colleagues who are women tend to be less aggressive about pursuing opportunities that may feel like a 'reach' based on their current experience/roles but that they may want or be qualified for. Titles and roles = advancement and this must have an impact. (M/Prof)

Supplemental Table 15 (cont): Representative quotes related to survey themes and sub-themes

	Visibility of Accomplishments	I feel women's accomplishments are relatively less visible than men's, so I may perceive women as less academically accomplished compared with their male peers. Visible achievements are often by men in the department, and the degree of career success that achievement promotes is more visible. (F/Asst)<br><br>I was told by my direct supervisor that, “there is no difference” between myself and another faculty member that just graduated and was hired out of fellowship. I have been practicing for over 10 years. I doubt that this mentality and statement would have been said to my male colleague who has the same experience as me. Furthermore, I’ve been told that I’m not “qualified” to [obtain a leadership position], yet a male counterpart who is my significant junior … is qualified and [was chosen for the position]. (F/Asst)<br><br>I feel that being an Asian-American female in anesthesia makes me feel that I have to work a little harder to network/be noticed/receive career advancement opportunities not of my own creation than my white male or female colleagues. (F/Assoc)<br><br>As a woman, I have always felt like I have to work just a bit or a lot harder to be taken seriously and to receive similar resources as my male counterparts. (F/Prof)
	Uncompensated Work	The disproportionate amount of unpaid invisible labor. (F/Asst)<br><br>My husband never cares about this kind of thing [childcare]. (F/Asst)<br><br>I’m the default care giver for all urgent/emergent matters. (F/Asst)<br><br>My husband is incredibly supportive and a capable parent but there are still many roles related to parenting/caregiving that fall to me by default as well as by choice. I am the more administratively minded parent in many ways so even if my husband can take care of details like buying tickets for the kids' dance recitals, buying new uniforms, finding teachers/studios for their extracurricular activities, often if I'm not delegating/reminding him of certain tasks or taking care of them on my own, then balls will get dropped. I'm not sure why but I feel like moms care a lot more about when balls get dropped than dads do, even though it affects the children equally no matter who actually dropped the ball. (F/Assoc)<br><br>From my earlier experience, the expectations at work are same for both genders whereas the expectation at home is significantly more for the woman- probably across cultures and continents. (F/Assoc)<br><br>As a female in the department, I often take on extra responsibilities like event planning (celebrations) and supporting colleagues during significant life events, which go uncompensated compared to weekend and night work which are heavily compensated. I am asked to handle celebrations/event planning but there is no compensation associated with it, even though it is a significant amount of work and not necessarily work I want to do. I would much prefer to do clinical work. [F/Assoc]<br><br>I perceive a significant need for off-hours work to have academic productivity and career success (need to fill roles that require off-hours work or need to use off-hours time for research or education) and I perceive that men have an easier time using that off-hours time (perhaps less responsibilities outside the workplace, such as childcare). (M/Asst)<br><br>My wife takes on many more of the home responsibilities, in addition to her work as a full-time physician. (M/Assoc)

Supplemental Table 15 (cont): Representative quotes related to survey themes and sub-themes

Work Culture	Operating Room Culture	There is no community. There is very little incentive for people to work together or to be efficient. [First case on time] start is a useless metric. Have more discussions with surgeons/OR staff about productivity. Incentivize productivity. (F/Asst)<br><br>There are CONSTANT microaggressions and having to placate egos as well as accept egregious behavior that goes unchecked and un-noticed by management. It's taking responsibility for other peoples' lack of understanding and choosing to constantly forgive rudeness, offensive behavior and ignorance. (F/Asst)<br><br>I have also experienced and/or witnessed micro and macroaggressions due to my race and gender. (F/Assoc)<br><br>As a confident tall white male, my personal identity has a strongly positive impact on my experience, even as an early career anesthesiologist. (M/Asst)<br><br>Middle-aged white male = automatic assumption of authority. (M/Assoc)<br><br>Work on some of the toxic culture in the ORs that I know impacts women more significantly than men. (M/Prof)
	Alignment Between Individual and Institutional Values	A lot of academic/faculty work seems to be dependent on the generosity of faculty members, while the health system wants to work their clinicians as though this is a private practice and we have no professional responsibilities aside from patient care. Hence the private practice drain. If the health system prioritized academic work as much as it does clinical billing, it would be much easier for both men AND women to find joy in their academic position." (F/Asst)<br><br>Misogyny, microaggression, inequality … when [the institution] touts that it is committed to DEI. (F/Asst)<br><br>Communication, representation at leadership level. (F/Asst)
	Clinical Schedule	Allow more flexibility in the timing of shifts, schedules. Either earlier leave times or later leave times. Allow for partial shifts. (F/Asst)<br><br>The unpredictability of the schedule and work compression are constant stressors. (F/Asst)<br><br>A few negatives are inflexible scheduling… daily time unpredictability, and no location preference for working sites. If I could work at the locations closer to my house, I might be able to spend more time with my kids, I waste almost 1.5-2 hours in commute due to traffic vs 20-45 minutes. This is purely based on the proximity of certain sites. (F/Assoc)<br><br>The agency over one's life is a determinant of wellbeing for humans in general. Adequate staffing, reasonable workload and predictability should be the same for all. ... I think having caring principles, transparency and equity for all will benefit each member in the department individually (F/Prof)<br><br>Improve the factors that drive burnout: understaffing, lack of control re hours, unpredictability and late notice of OR allocations and finish times, lack of respect from (many but not all) surgeons from most surgical sub-specialties (M/Assoc)<br><br>Our scheduling process … does not confer the same flexibility that exists in other specialties or professions. much of this is our own failure to reimagine how these workflows can be managed... (M/Prof)<br><br>Redesign the entire scheduling process from the ground up to enhance flexibility and to reframe the job of the schedulers to help faculty. (M/Prof)

Supplemental Table 15 (cont): Representative quotes related to survey themes and sub-themes

	Pay and Non-clinical Time Transparency	Poor transparency into the workings of compensation. (F/Asst)<br><br>Unequal distribution of opportunities for leadership and advancement. Misogyny. (M/Asst)<br><br>[Improving] transparency in pay, distribution of shifts [would improve the work environment]. (M/Asst)
Work-Life Integration	Parenting	Juggling pregnancy, multiple childbirths, breastfeeding, childcare responsibilities, and managing children's medical needs while also being the primary decision-maker when they're sick is demanding. Despite having a full-time nanny with flexible hours, handling sick children's doctor visits falls on me. Balancing weekend and night shifts for professional growth and compensation is tough with young children and breastfeeding commitments. (F/Assoc)<br><br>Misalignment of daycare/school hours with clinical responsibilities is a humongous problem (less so for a mostly-researcher); literally could not contribute to drop-off or pickup on 80% of clinical days, which forces an imbalance in responsibilities that women "pay for" in other ways. (F/Assoc)<br><br>Picking them up. Get some quality time after work, prepare dinner and bring them to bed. (F/Assoc)<br><br>Mom guilt is real- you can never feel like you're succeeding at something without failing or sacrificing something else (F/Assoc)<br><br>The unpredictability of our schedule from day to day, week to week, and month to month make it IMPOSSIBLE to reliably bring kids to activities, practices, games, swimming, etc. This is also stressful because we are rarely the parents who offer carpools/rides, etc given the unpredictable timing of when we finish in the OR, etc. This has required outsourcing and also means we often miss important events in our children's lives. (F/Assoc)
	Compensation	Increased salary and increased resources for childcare. (F/Assoc)<br><br>Sick days— often we are sick and taking care of a sick child but there are no sick days and the kid cannot go to day care sick. So you are losing money at work and at daycare. (F/Assoc)<br><br>Compensation [is a threat to retention]. (M/Asst)<br><br>High cost of living requiring high workload for extra compensation. (M/Prof)
	Personal Wellness	Longer work hours, night calls, less flexibility to take off at work, no allotted sick days. (F/Asst)<br><br>Lack of time to pump if breastfeeding, the lack of ramp up time when returning to work after maternity leave… (F/Assoc)<br><br>Trying to do everything and be everything when that is impossible. (F/Assoc)<br><br>Lack of sleep [is a major problem for new parents]. (M/Asst)<br><br>Work life balance [is a threat to retention]. (M/Prof)
F, female; M, male, Asst, assistant professor, Assoc, associate professor, Prof, professor; OR, operating room; DEI, diversity, equity, inclusion		

Supplemental Figure 15:
Potential Interventions to Address Career Advancement Sub-themes

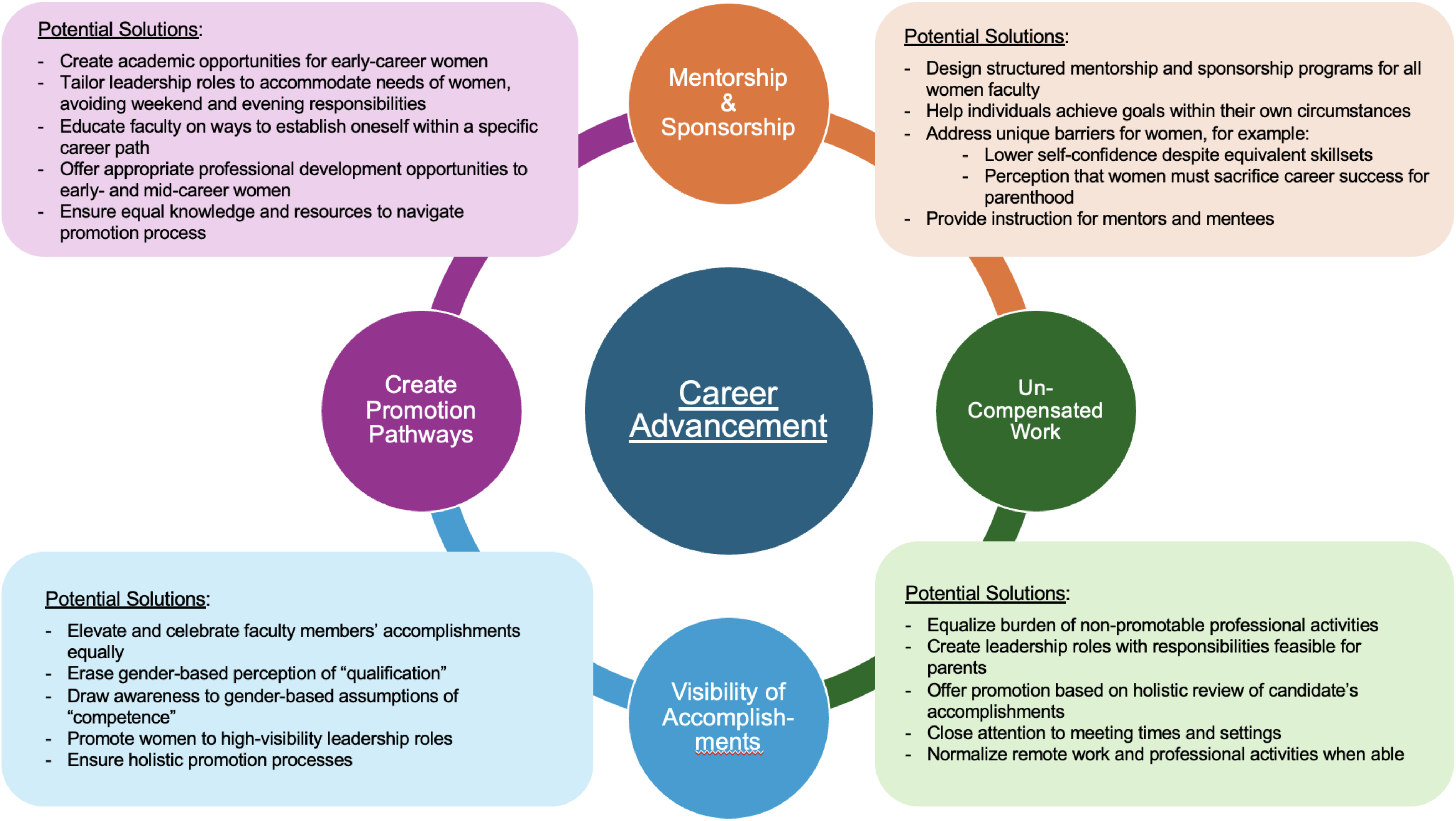

Supplemental Figure 16: Potential Interventions to Address Work Culture Sub-themes

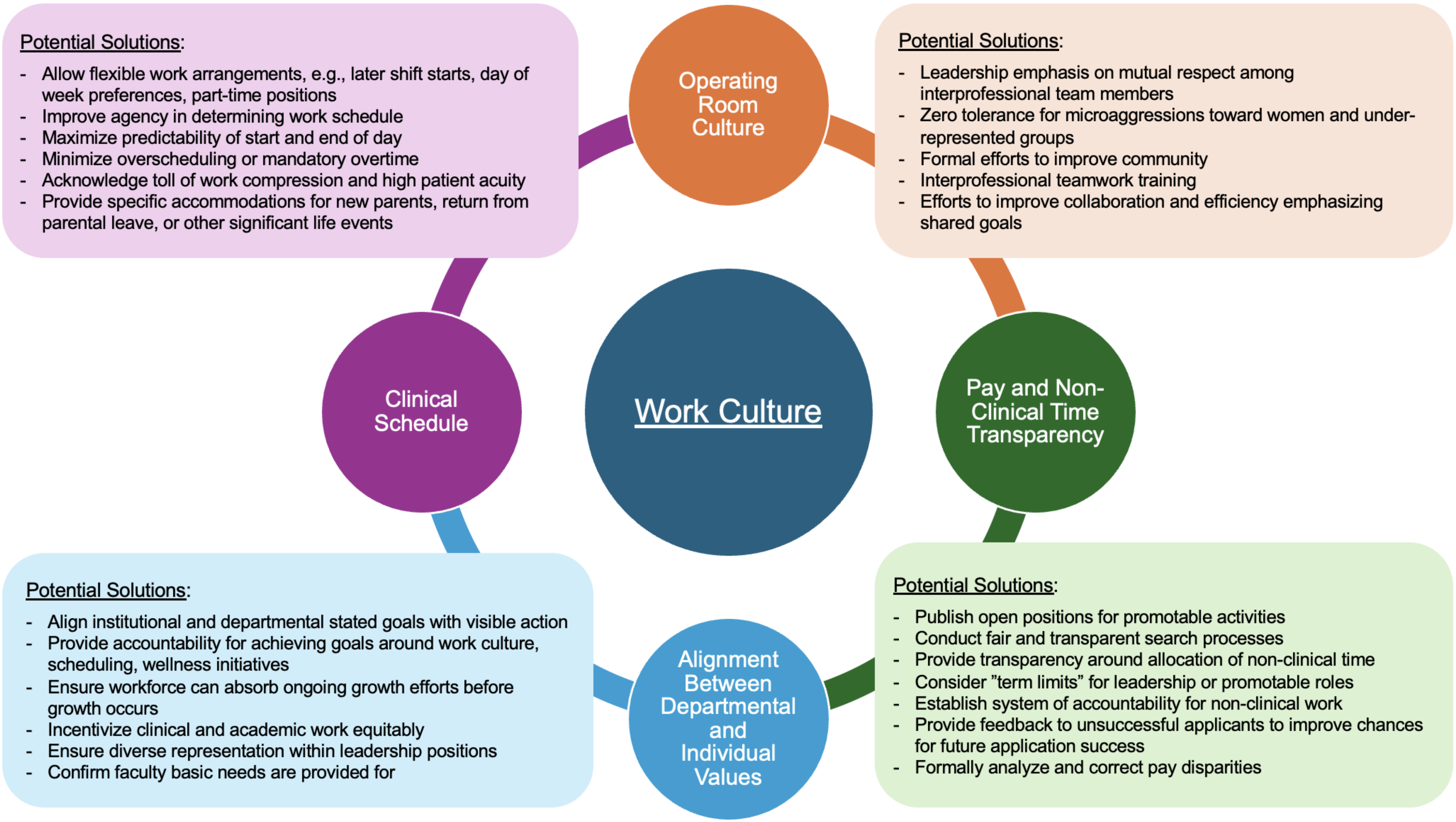

Supplemental Figure 17:
Potential Solutions for Providing Balance between Home and Work for Parents

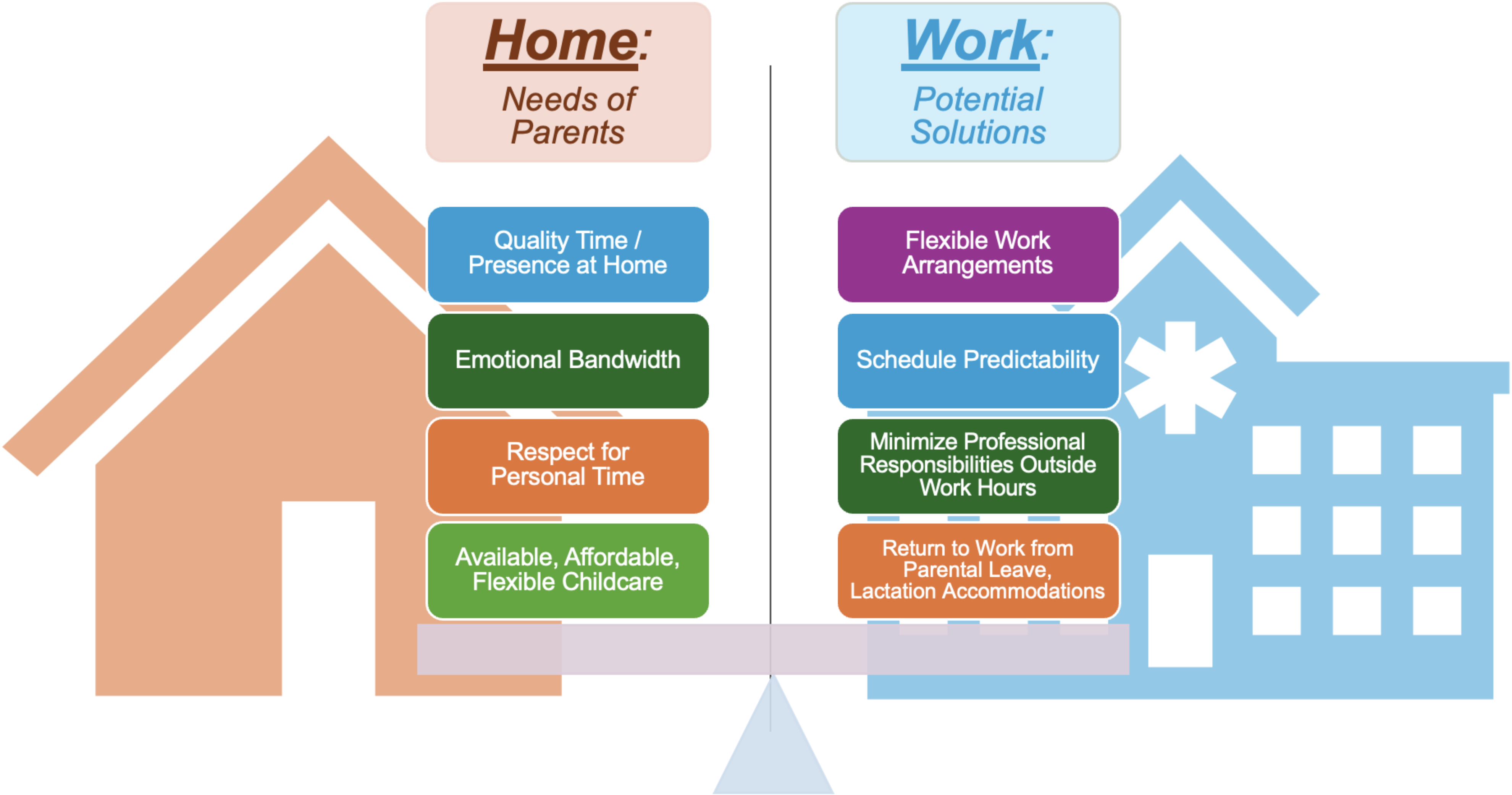

Supplement: Supplementary file 2 — Supplementary Material 2: Supplemental Document 1: Detailed Survey Results. Quantitative Survey Results. Supplemental Figure and Table 1: Academic rank by years on faculty. Supplemental Figure and Table 2: Department leadership positions by years on faculty. Supplemental Figure and Table 3: Institutional leadership positions by years on faculty. Supplemental Figure and Table 4: National leadership positions by years on faculty. Supplemental Figure and Table 5: Ever served on an editorial board by years on faculty. Supplemental Figure and Table 6: Number of publications in the past 5 years by years on faculty. Supplemental Table 6.1: 0-2 vs. 3+ publications by years on faculty. Supplemental Figure and Table 7.1 and 7.2: Research funding by years on faculty. Supplemental Figure and Table 8: Number of Departmental Awards by Years on Faculty. Supplemental Figure and Table 9: Number of National Awards by Years on Faculty. Supplemental Figure and Table 10: Non-promotable activity by years on faculty. Supplemental Table 11: Allies, Mentors, and Sponsors. Supplemental Table 12: Reasons for Leaving or Staying. Supplemental Figure 12: Primary parent. Supplemental Table 13: Sources of stress for parents. Supplemental Figure 13: Parenting stress differences between men and women. Supplemental Figure and Table 14: Impostor phenomenon. Qualitative Survey Results. Supplemental Table 15: Representative quotes related to survey themes and sub-themes. Supplemental Figure 15: Potential Interventions to Address Career Advancement Sub-themes. Supplemental Figure 16: Potential Interventions to Address Work Culture Sub-themes. Supplemental Figure 17: Potential Solutions for Providing Balance between Home and Work for Parents [file 12871_2025_3522_MOESM2_ESM.pdf]
