# Supplementary material for: Understanding professional disparities in academic anesthesiology: a single-center gender-based survey study
Source: BMC Anesthesiol. 2025 Dec 7;26:31. doi: 10.1186/s12871-025-03522-z (PMC12797490; doi:10.1186/s12871-025-03522-z)
Supplement: Supplementary file 3 — Supplementary Material 3: Supplemental Document 2: Codebook [file 12871_2025_3522_MOESM3_ESM.docx]

**Table 1: Coding guide**

| **Career Advancement** | **Work Culture** | **Work-Life Integration** |
| --- | --- | --- |
| Mentorship/  sponsorship program | Perception of qualification | Parenting* |
| Leadership representation | Uncompensated roles | Cost of living |
| Feasible leadership opportunities | Microaggressions | Compensation |
| Impostor phenomenon | Automatic privilege (males) | Schedule * |
| Investment in women | Poor teamwork | Work compression |
| Sponsor women into leadership | Disrespect | Personal needs |
| Promotion equity | Lack of community |  |
|  | Institutional priorities* |  |

* Has sub-codes

**Table 2: Sub-codes**

|  |
| --- |
| \| **Parenting** \| **Institutional Priorities** \| **Schedule** \| \| --- \| --- \| --- \| \| Sacrifice academic productivity \| Growth \| Need for flexibility \| \| Desire for presence \| Diversity, equity, inclusion \| Need for predictability \| \| Childcare cost, availability \|  \| Minimize over-scheduling \| \| Return to work from leave \|  \|  \| \| Lactation accommodations \|  \|  \| \| Female-dominant issue \|  \|  \| |
|  |
|  |
